# The Mediator complex subunit MoMed15 plays an important role in conferring sensitivity to isoprothiolane by modulating xenobiotic metabolism in *M. oryzae*

**DOI:** 10.1128/mbio.01778-24

**Published:** 2024-11-12

**Authors:** Fan-Zhu Meng, Wen-Kai Wei, Min-Zheng Cai, Zuo-Qian Wang, Liang-Fen Yin, Wei-Xiao Yin, Guido Schnabel, Chao-Xi Luo

**Affiliations:** 1 Hubei Key Lab of Plant Pathology, College of Plant Science and Technology, Huazhong Agricultural University, Wuhan, China; 2 Institute of Plant Protection and Soil Science, Hubei Academy of Agricultural Sciences, Wuhan, China; 3 Experimental Teaching Center of Crop Science, College of Plant Science and Technology, Huazhong Agricultural University, Wuhan, China; 4 Department of Plant and Environmental Sciences, Clemson University, Clemson, South Carolina, USA; Cornell University, Ithaca, New York, USA

**Keywords:** *Magnaporthe oryzae*, isoprothiolane resistance, Mediator complex subunit, xenobiotic metabolism

## Abstract

Rice blast caused by *Magnaporthe oryzae* is one of the most economically important rice diseases. Fungicides such as isoprothiolane (IPT) have been used extensively for rice blast control, but resistance to IPT in *M. oryzae* is an emerging threat. In this study, molecular mechanisms of resistance in IPT-resistant mutants were identified. Through whole-genome sequencing and genetic transformation, we identified the gene *MoMed15*, encoding a transcriptional glutamine-rich co-activator Mediator complex subunit, in which mutations or deletion resulted in moderate IPT resistance. Further research found that MoMed15 physically interacted with the IPT resistance regulatory factor MoIRR to simultaneously regulate both MoIRR expression and the expression of multiple xenobiotic-metabolizing enzymes in response to IPT stress. We hypothesize that some xenobiotic-metabolizing enzymes enhance IPT toxicity by modifying the IPT structure. Variation of MoMed15 affected the recruitment of the transcriptional Mediator complex and decreased the expression of these xenobiotic-metabolizing enzymes, resulting in moderate IPT resistance. We also found that *MoPGR1,* encoding a protein that activates cytochrome P450 enzymes, was essential to confer IPT sensitivity, and its expression was directly regulated by MoIRR.

**IMPORTANCE:**

Isoprothiolane (IPT) has been used extensively for the management of rice blast disease and IPT-resistant subpopulations have emerged in Chinese rice fields. The emergence of resistant pathogen populations has led to a steep increase in fungicide use, increasing pesticide risk for the applicator and the environment. The molecular mechanisms of IPT resistance in *M. oryzae* remain elusive. In this study, we demonstrated that transcriptional co-activator MoMed15 interacts with IPT resistance regulator MoIRR to recruit the Mediator complex, which promotes the expression of xenobiotic-metabolizing enzymes, leading to exacerbated IPT toxicity. The *MoMed15* could be used for IPT resistance detection in rice fields.

## INTRODUCTION

Rice blast caused by the ascomycete fungus *Magnaporthe oryzae* B.C. Couch causes damage to rice crops worldwide (1). The occurrence of rice blast can lead to a 10%–30% reduction in rice production annually, and in severe cases, it may cause large-scale yield loss at harvest (2). Isoprothiolane (IPT) is a systemic fungicide that was developed in the 1970s for control of rice blasts based on its inhibitory effect on mycelial growth and infection peg formation of *M. oryzae*. The mode of action of IPT is still unclear, but it may interfere with transmethylation in the biosynthesis of phosphatidylcholine (PC) (3, 4). It also is used as an insecticide to control rice planthoppers and as a plant growth regulator to prevent damping-off disease caused by a nonparasitic physiological disorder in rice nurseries (5, 6).

Long-term use of fungicides with site-specific modes of action can lead to the emergence and selection of resistance in plant pathogens, which, in turn, can result in control failure (7). There are four main molecular mechanisms of fungicide resistance: mutations in the target gene, overexpression of the target gene, enhanced efflux pump activity of fungicides, and enhanced cellular metabolic capacity (8). For example, mutations of glutamic acid at position 198 (198A/G/K/V) and phenylalanine at position 200 (F200Y) in the β-tubulin caused resistance to benzimidazole fungicides in *Botrytis cinerea* (9). Overexpression of *MfCYP51* due to an upstream insertion sequence named “Mona” caused resistance to sterol demethylation inhibitor (DMI) fungicides in *Monilinia fructicola* (10, 11). Overexpression of ABC (ATP-binding cassette) transporter *atrB* and major facilitator superfamily transporter *mfsM2* genes resulted in multidrug resistance to fungicides in *B. cinerea* (12). And finally, overexpression of cytochrome P450 genes *ShCYP561, ShCYP65*, and *ShCYP68* led to multifungicide resistance in *Sclerotinia homoeocarpa* (13).

Due to the long-term use of IPT, resistant isolates were reported from several rice-growing areas in China (14), but the mechanism of resistance is still unknown. In laboratory mutants, Zn_2_Cys_6_ family transcription factor MoIRR and velvet family transcription factor MoVelB were associated with a moderate and low resistance to IPT, respectively (15–17).

The transcriptional cofactor Mediator complex plays a key role in global gene regulation in eukaryotes (18). Med15 is one of the subunits of the Mediator complex, which usually interacts with the transcriptional activation domain of transcription factors to promote the expression of downstream target genes (19). Transcription factors recruited to Mediators play important roles in regulating the expression of genes in drug resistance, stress tolerance, growth and development, and virulence in fungi, plants, and mammals (20–26). For example, under azole fungicide stress, the transcription factor ScPDR1 in *Saccharomyces cerevisiae* recruits a Mediator complex and then interacts with ScMed15, thereby activating expression of the downstream ABC transporter protein PDR5 to cause azole resistance (20).

In this study, *MoMed15* was found to be associated with IPT resistance. We confirmed that MoMed15 interacts with MoIRR to recruit the Mediator complex and activates the expression of downstream xenobiotic-metabolizing genes. We also demonstrated that the gene *MoPGR1*, encoding a protein that activates the cytochrome P450 enzymes, is a key downstream target of MoIRR, but its expression was not dependent on the assistance of MoMed15. This study identified a novel regulatory mechanism for IPT resistance and provides a theoretical basis for designing new fungicides for rice blast control.

## RESULTS

### Identification of loci conferring resistance to IPT in *M. oryzae*

**Fig 1:**
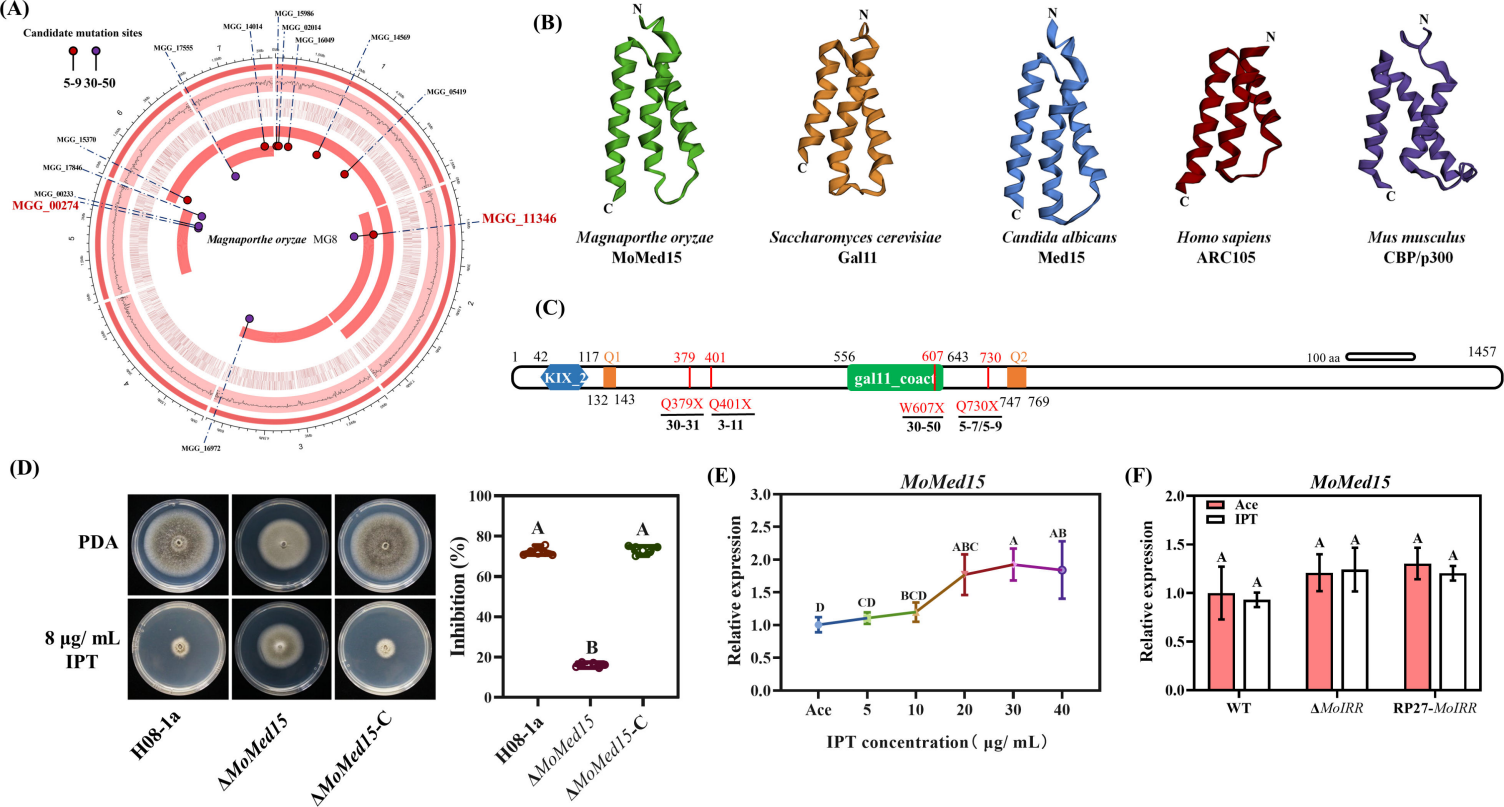
MoMed15 was associated with the resistance to IPT in *M. oryzae*. (**A**) Identification of SNPs and InDels in 5-9 and 30-50 mutants by whole-genome sequencing. Using the wild-type H08-1a genome as a control, the SNPs and InDels were analyzed in the 5-9 and 30-50 mutants using Lofreq software (version 2.1.5). (**B**) The 3D structure of KIX domain in *Magnaporthe oryzae*, *Saccharomyces cerevisiae*, *Candida albicans*, *Homo sapiens,* and *Mus musculus*. Prediction of 3D protein structures was done using the website https://robetta.bakerlab.org/. (**C**) Analysis of MoMed15 mutation preference by Sanger sequencing of *MoMed15* gene in IPT-resistant mutants. (**D**) Knockout transformants Δ*MoMed15* exhibited moderate resistance to IPT. A 3-mm mycelial plug of each strain was inoculated on PDA or PDA amended with 8 µg/mL IPT and then incubated at 27°C for 5 days (left panel), and the mycelial growth inhibition was calculated for each strain (right panel). (**E**) Expression of *MoMed15* in wild-type isolate H08-1a at different concentrations of IPT. Each strain was cultured in PDB for 48 h and then treated with 0, 5, 10, 20, 30, and 40 µg/mL IPT for 3 h, respectively. (**F**) Expression of *MoMed15* in different strains with or without IPT. Each strain was cultured in PDB for 48 h and then treated with 10 µg/mL IPT or acetone for 3 h, respectively. The *MoActin* gene was used as the internal reference for normalization. The data presented are the mean ± SD (*n* = 3). Bars followed by the same letter are not significantly different according to a least significant difference test at *P* = 0.01.

In the initial investigation, a total of 77 resistant mutants were generated on IPT-amended potato dextrose agar (PDA) with wild-type strain H08-1a (17). Some grew slower, produced less melanin, lost hydrophobicity, and were unable to produce conidia (see Fig. S1B in the supplementary material). To identify the genes associated with IPT resistance in these mutants, we performed whole-genome sequencing and single nucleotide polymorphism (SNP) or InDel analysis in two representative mutants 5-9 and 30-50. We identified 8 SNPs in the 5-9 mutant, 5 SNPs and 1 InDel in the 30-50 mutant, and mutations in *MGG_11346* in both mutants (Fig. 1A; see Table S3 in the supplementary material). *MGG_11346* encodes a protein containing the KIX and gal11_coact structural domains. Amino acid comparison and three-dimensional (3D) protein structure analysis of the KIX domain in *M. oryzae* MGG_11346, *S. cerevisiae* Gal11, *C. albicans* Med15, human ARC105, and mouse CBP/P300 showed that the structure of MGG_11346 was similar to Gal11, Med15, ARC105, and CBP/P300 in terms of KIX amino acid sequence and 3D structure (Fig. 1B; see Fig. S1C in the supplementary material). Phylogenetic analysis also showed that MGG_11346 was conserved in phytopathogenic fungi (see Fig. S1D in the supplementary material), and thus we named this protein MoMed15.

All five mutants revealed mutations in *MoMed15* (Fig. 1C). Specifically, mutant 3-11 had changed at position 401 from codon CAG (Gln) to the stop codon (TAG); mutants 5-7 and 5-9 had a variation at position 730, changing the codon CAG (Gln) to the stop codon (TAG); and mutants 30-31 and 30-50 had the stop codon TAG at position 379 (from CAG) and 607 (from TGG), respectively (Fig. 1C). Interestingly, there were variations between strain H08-1a and the GenBank deposited strains 70–15 and Y34 (see Fig. S1E in the supplementary material). Among the identified *MoMed15* mutations from IPT-resistant mutants, the codon preference encoding glutamine was mutated to a stop codon (Q/X), leading to premature termination of translation of MoMed15, and the formation of a shorter and nonfunctional protein.

To further clarify the involvement of *MoMed15* in the regulation of IPT resistance in *M. oryzae*, we obtained *MoMed15* knockout transformants and corresponding complemented transformants (see Fig. S2A and B in the supplementary material). The Δ*MoMed15* mutant was less sensitive to IPT compared to wild-type H08-1a, and the complemented transformant Δ*MoMed15*-C restored the Δ*MoMed15*-deficient phenotypes (Fig. 1D). By contrast, the overexpression transformant OE*MoMed15* did not show significant differences in the sensitivity to IPT, compared to the wild-type strain H08-1a (see Fig. S2C in the supplementary material). The above results suggest that mutations leading to loss of function, rather than overexpression of *MoMed15,* were responsible for the acquisition of IPT resistance in *M. oryzae*.

To clarify whether *MoMed15* expression was induced or suppressed by IPT treatment, mutants were subjected to different concentrations of IPT. As shown in Fig. 1E, the expression level of *MoMed15* was not affected by increasing concentrations of IPT. Meanwhile, the expression of *MoMed15* was not regulated by MoIRR, a transcription factor that negatively regulates IPT resistance in MR-2 type of mutants, indicating *MoMed15* is not a downstream target gene of MoIRR (Fig. 1F).

### MoMed15 mutations impacted the pathogenicity of *M. oryzae*

Compared to the wild-type strain H08-1a, both Δ*MoMed15* transformants and the *MoMed15* mutants exhibited IPT resistance, but there were significant differences in their resistance levels (see Fig. S3A and B in the supplementary material). To further evaluate whether Δ*MoMed15* transformants and the *MoMed15* mutants showed fitness costs, growth rate, spore production, appressorium formation rate, hydrophobicity, environmental stress tolerance, and virulence were investigated. Δ*MoMed15* transformants and the *MoMed15* mutants exhibited slower growth, did not produce conidia or appressoria, and lost hydrophobicity compared to wild-type strain H08-1a (see Fig. S3C through F in the supplementary material). Although Δ*MoMed15* transformants and *MoMed15* mutants did not show obvious differences in tolerance to glycerol, NaCl, sorbitol, and sodium dodecyl sulfate (SDS; see Fig. S3G in the supplementary material), their virulence was significantly decreased (see Fig. S3H in the supplementary material). The above results indicate that MoMed15 is an indispensable virulence factor.

### MoMed15 mutants were resistant to IPT and IBP, but not to TEB

The Δ*MoMed15* transformants and *MoMed15* mutants were subjected to PDA amended with iprobenfos (IBP), tebuconazole (TEB), fludioxonil (FLU), iprodione (IPR), rapamycin (RAP), and carbendazim (CAR). As shown in Fig. S4A and B, Δ*MoMed15* transformants and *MoMed15* mutants were less sensitive to IBP but more sensitive to TEB. The effect of other fungicides was generally not different between H08-1a and transformants/mutants (see Fig. S4B in the supplementary material). MoMed15 mutants were cross-resistant to IPT and IBP (ρ = 0.864, *P* < 0.01), but analysis of sensitivity to fungicides with different modes of action revealed that the sensitivity of these mutants was negatively correlated between IPT and TEB (ρ= ‒ 0.638, *P* < 0.01). Only low to moderate correlations were observed for other fungicides (see Fig. S4C in the supplementary material).

### MoMed15 and MoIRR were both involved in regulating the sensitivity to IPT in *M. oryzae*

**Fig 2:**
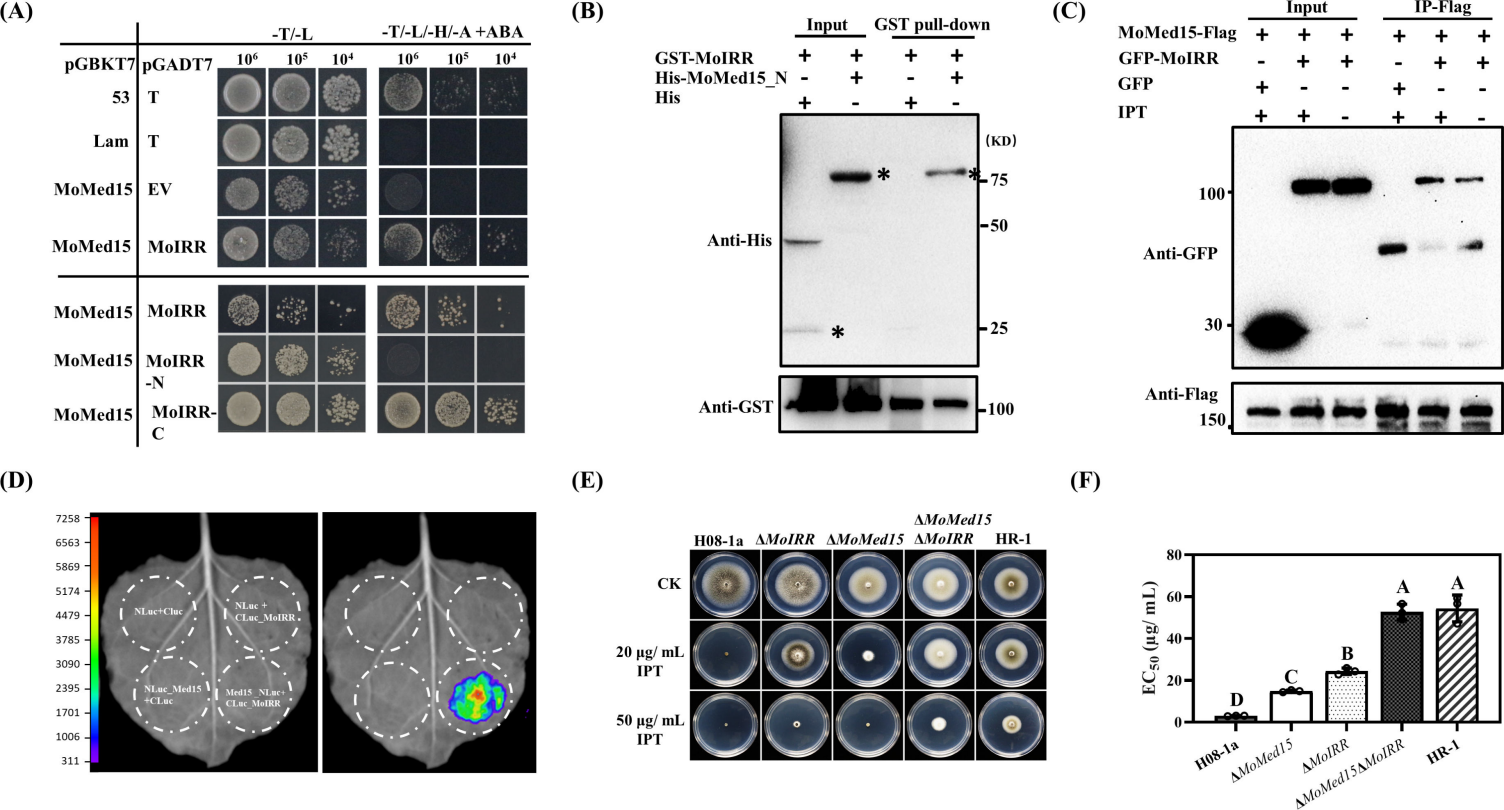
MoMed15 and MoIRR were jointly involved in IPT sensitivity in *M. oryzae*. (**A**) Verification of interaction between MoIRR and MoMed15 by yeast two-hybrid assay. pGBKT7-53 and pGADT7-T were used as positive control, pGBKT7-Lam and pGADT7-T as negative control. (**B**) GST pull-down assay demonstrated that MoIRR interacted with the N-terminus of MoMed15. His-MoMed15-N but not His was pulled down by GST-MoIRR. The input and pull-down proteins were detected with anti-His and anti-GST antibodies (target bands were indicated with the symbol ‘*’). (**C**) Co-IP analysis was used to detect the interaction between MoIRR and MoMed15 in the mycelium. The input and IP-Flag proteins were detected with anti-Flag and anti-GFP antibodies. Each strain was cultured in PDB for 48 h, and then treated with 10 µg/mL IPT or acetone for 3 h, respectively. (**D**) Luciferase complementation assay was used to detect the interaction between MoIRR and MoMed15 in *N. benthamiana* leaves. cLUC_MoIRR and nLUC_MoMed15 were co-expressed in *N. benthamiana* leaves *via Agrobacterium*-mediated transient expression. The luminescence signal was observed at 48 h after infiltration. (**E**) Detection of the IPT sensitivity of different types of transformants or mutants of *MoMed15* and *MoIRR*. A 3-mm mycelial plug of each strain was inoculated on PDA or PDA amended with 20, 50 µg/mL IPT and then incubated at 27°C for 5 days. (**F**) Statistical analysis of the EC_50_ values in different types of transformants or mutants of *MoMed15* and *MoIRR*. The data presented are the mean ± SD (*n* = 3). Bars followed by the same letter are not significantly different according to a least significant difference test at *P* = 0.01.

The Zn_2_Cys_6_ transcription factor MoIRR is crucial in the regulation of IPT resistance in *M. oryzae*. To clarify whether MoIRR recruits the Mediator complex and activates the downstream target genes, we investigated the interaction between the MoIRR and Med15 *via* yeast two-hybrid assay (Fig. 2A; see Fig. S5A and B in the supplementary material). The results showed that the C-terminus of MoIRR containing the activation domain interacted with MoMed15 (Fig. 2A). On the other hand, the ABD (helical activator-binding domains) portion of MoMed15 interacted with MoIRR (see Fig. S5B in the supplementary material). Meanwhile, GST pull-down assay demonstrated that MoIRR interacted with MoMed15 (Fig. 2B). Also, MoIRR and MoMed15 interacted *in vivo* by co-immunoprecipitation (Co-IP) and luciferase complementation assay (LCA) (Fig. 2C and D). The double knockout transformant Δ*MoMed15*Δ*MoIRR* exhibited high resistance to IPT, similar to the *MoIRR* and *MoMed15* double mutated strain HR-1 (Fig. 2E and F).

### MoMed15 and MoIRR regulated the expression of genes related to xenobiotic metabolism

**Fig 3:**
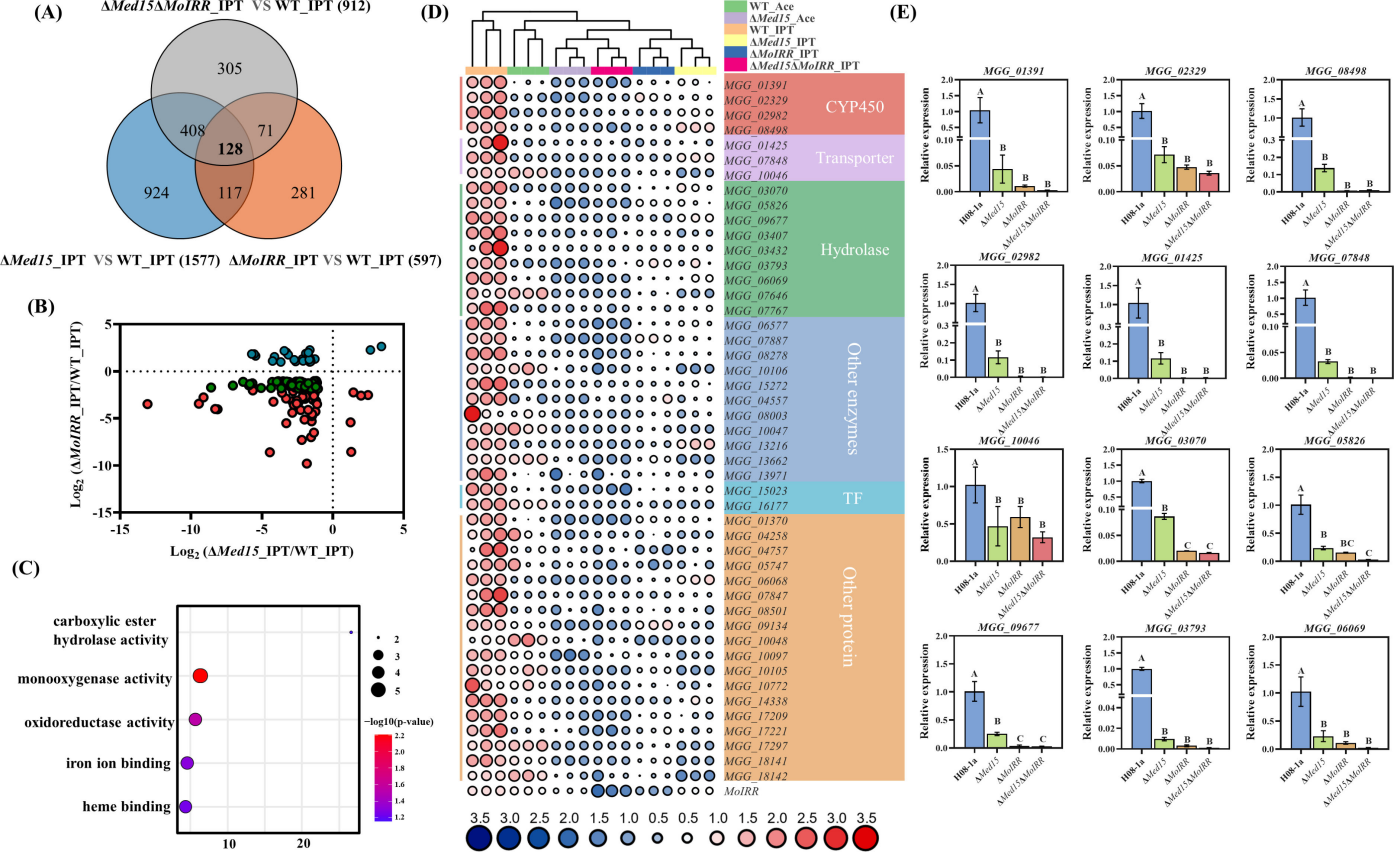
MoMed15 and MoIRR regulated the expression of xenobiotic metabolism genes to determine the IPT sensitivity. (**A**) Analysis of co-differentially expressed genes (log2 fold change ≥1 or ≤ −1 and *P* value of ≤ 0.05) in Δ*MoMed15*, Δ*MoIRR,* and Δ*MoMed15*Δ*MoIRR* transformants with IPT treatment. (**B**) Volcano plot analysis of highly differential DEGs (log2 fold change ≥1.5 or ≤−1.5 and *P* value of ≤ 0.05) in Δ*MoMed15* and Δ*MoIRR*, compared to wide-type H08-1a. (**C**) Gene ontology enrichment analysis of DEGs (log2 fold change ≥1.5 and *P* value of ≤ 0.05) downregulated in different knockout transformants compared to H08-1a under 25 µg/mL IPT treatment for 4 h. (**D**) Heat map displaying the expression and functional annotation of downregulated DEGs under different conditions. (**E**) Reverse transcription PCR (RT-PCR) verification of the expression of the xenobiotic metabolism genes encoding CYP450 proteins, ABC transporter proteins, and hydrolase proteins in Δ*MoMed15*, Δ*MoIRR*, and Δ*MoMed15*Δ*MoIRR* under 25 µg/mL IPT treatment for 4 h. The *MoActin* gene was used as the internal reference for normalization. The data presented are the mean ± SD (*n* = 3). Bars followed by the same letter are not significantly different according to a least significant difference test at *P* = 0.01

To further investigate the mechanism by which MoMed15 and MoIRR regulate IPT sensitivity, we performed RNA-seq on wild-type H08-1a, Δ*MoMed15*, Δ*MoIRR,* and Δ*MoMed15*Δ*MoIRR* transformants treated with 25 µg/mL IPT for 4 h. A total of 128 differentially expressed genes (DEGs) were obtained based on the comparison of the transcriptomic profiles of Δ*MoMed15*, Δ*MoIRR,* and Δ*MoMed15*Δ*MoIRR* transformants to wild-type H08-1a under IPT treatment (Fig. 3A). As shown in Fig. 3B, 98 DEGs exhibited downregulated expression in Δ*MoMed15* and Δ*MoIRR* transformants compared to wild-type H08-1a, consistent with the function of MoMed15 as a transcriptional coactivator. Gene ontology (GO) enrichment analysis showed that monooxygenase activity and carboxylic ester hydrolase activity were significantly enriched in the downregulated DEGs (Fig. 3C).

Drug metabolizing enzymes play a central role in the conversion, metabolism, or detoxification of xenobiotic compounds that enter the cells. Heat map illustrated expression levels and functional annotations of 48 downregulated DEGs in Δ*MoMed15* and Δ*MoIRR* RNA-seq data (Fig. 3D). In particular, some xenobiotic metabolism genes, for example, phase I metabolizing enzymes (cytochrome P450s, Hydrolase) and the phase III secretion system, ATP-binding cassette (ABC) transporter proteins encoding genes were induced in wild-type isolate H08-1a under IPT treatment; however, the expression of these genes was significantly decreased in Δ*MoIRR*, Δ*MoMed15,* or Δ*MoMed15*Δ*MoIRR*, suggesting that MoMed15 and MoIRR were involved in IPT resistance by regulating the expression of xenobiotic metabolism genes. We also confirmed these results by reverse transcription quantitative PCR (RT-qPCR), that is, four CYP450s (MGG_01391, MGG_02329, MGG_02982, and MGG_08498), three ABC transporter proteins (MGG_01425, MGG_07848, and MGG_10046), and five hydrolases (MGG_03070, MGG_05826, MGG_09677, MGG_03793, and MGG_06069) encoding genes were significantly downregulated in Δ*MoMed15*, Δ*MoIRR,* and Δ*MoMed15*Δ*MoIRR*, indicating positive regulation of xenobiotic metabolism genes by MoMed15 and MoIRR (Fig. 3E).

### MoMed15 enhanced the expression of *MoIRR* and other xenobiotic metabolism genes

**Fig 4:**
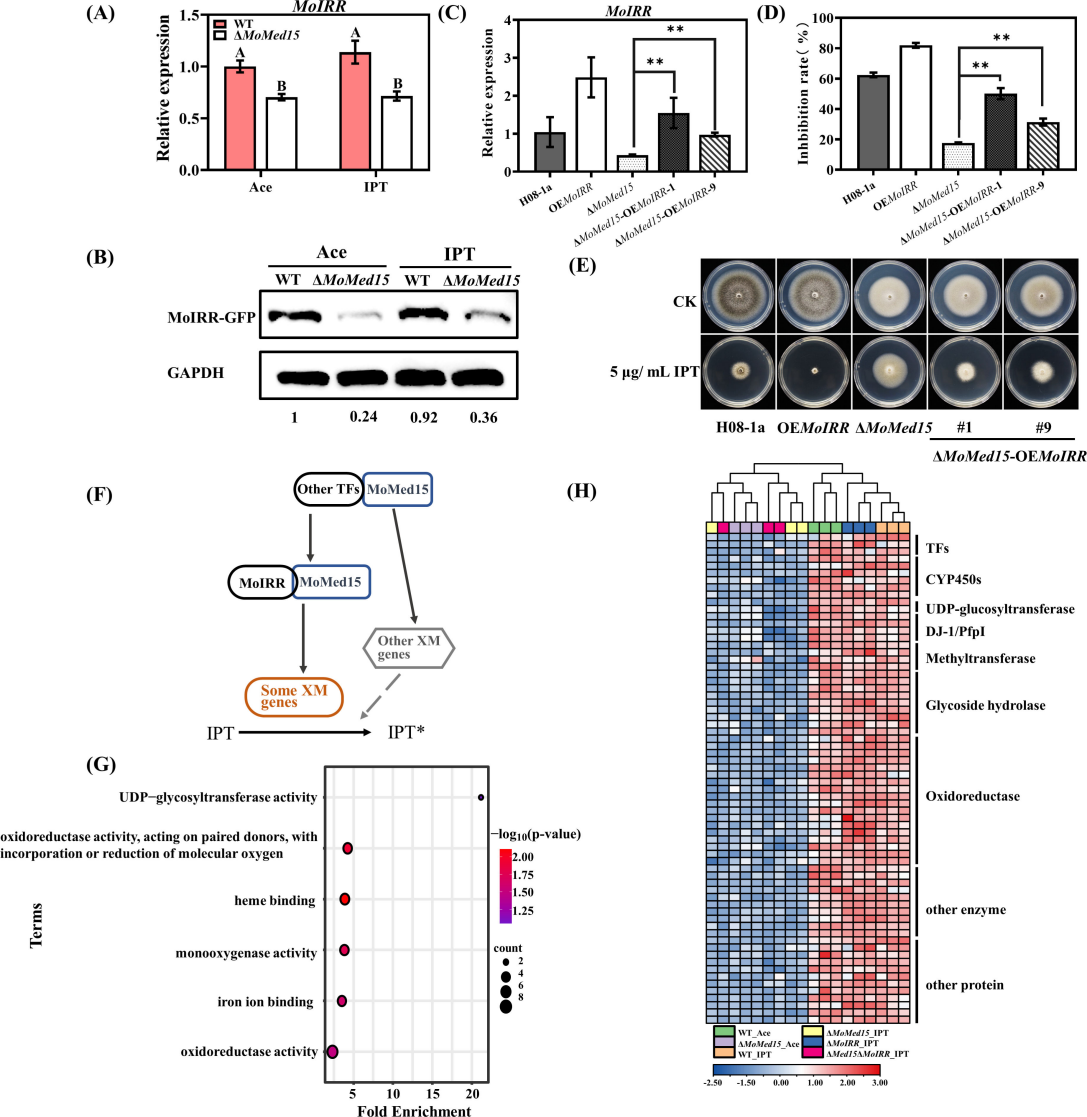
MoMed15 enhanced the expression of MoIRR and other xenobiotic metabolism genes. (**A**) Evaluation of *MoIRR* expression level in Δ*MoMed15* transformants by RT-qPCR with or without IPT. Ace: acetone, IPT: isoprothiolane. The *MoActin* gene was used as the internal reference for normalization. Each strain was cultured in PDB for 48 h, and then treated with 0 or 15 µg/mL IPT for 3 h, respectively. (**B**) The protein level of *MoIRR* in wild-type isolate H08-1a, *MoMed15* knockout transformants with or without IPT, GAPDH protein was used as an internal reference for Western blot detection. (**C**) Detection of *MoIRR* expression level in Δ*Med15*-OE*MoIRR* transformants by RT-qPCR. The *MoActin* gene was used as the internal reference for normalization. (**D**) Statistical analysis of the mycelial inhibition rate of the Δ*Med15*-OE*MoIRR* transformants under IPT stress. (**E**) Overexpression of *MoIRR* restored the sensitivity of *MoMed15* knockout transformants to IPT. The data presented are the mean ± SD (*n* = 3). Bars followed by the same letter are not significantly different according to a least significant difference test at *P* = 0.01. (**F**) A mode of regulation of MoMed15. (**G**) Gene ontology enrichment analysis of DEGs (log2 fold change ≥2 and *P* value of ≤ 0.05) downregulated in Δ*MoMed15*_IPT*,* Δ*MoMed15*Δ*MoIRR*_IPT, and no differentially expressed in the Δ*MoIRR*_IPT. (**H**) Heat map displaying the expression and functional annotation of downregulated DEGs that were regulated independently by *MoMed15*.

The expression level of *MoIRR* is one of the key factors in the regulation of sensitivity to IPT in *M. oryzae*. Surprisingly, we found that the expression of *MoIRR* had decreased in Δ*MoMed15*, compared to wild-type H08-1a in RNA-seq data. We further confirmed that the expression of *MoIRR* decreased in Δ*MoMed15* by RT-qPCR and Western blot, although fluctuation in the quantities of MoIRR protein was observed compared to mRNAs (Fig. 4A and B). We obtained Δ*MoMed15*-OE*MoIRR* transformants to clarify whether the reduction of *MoIRR* expression was critical for MoMed15 to regulate IPT resistance. The expression level of *MoIRR* in Δ*MoMed15*-OE*MoIRR* transformants had significantly increased compared to Δ*MoMed15* transformant (Fig. 4C). Accordingly, the mutants had partially restored sensitivity to IPT (Fig. 4D and E), indicating that MoMed15 affected the expression of MoIRR and sensitivity to IPT.

The Δ*MoMed15*Δ*MoIRR* double knockout transformants showed much higher resistance levels to IPT than Δ*MoMed15* and Δ*MoIRR*, and we speculate that MoMed15 still has other independent pathways to regulate IPT resistance (Fig. 4F). To further explore the regulatory network of MoMed15, we screened genes that were downregulated more than fourfold in the Δ*MoMed15*_IPT and Δ*MoMed15*Δ*MoIRR*_IPT samples but not differentially expressed in the Δ*MoIRR*_IPT samples, resulting in a total of 195 DEGs. GO and heat map analysis revealed that the expression of many xenobiotic metabolism genes, such as UDP-glycosyltransferase, CYP450, and hydrolase, was significantly decreased. That indicated that MoMed15 interacted with MoIRR to regulate the expression of some xenobiotic metabolism genes, and with yet unknown transcription factors to regulate the expression of other xenobiotic metabolism genes (Fig. 4G and H).

### IPT sensitivity was regulated by MoPGR1 in *M. oryzae*

The *MoMed15*-derived 30-50 and HR-1 mutants had similar basic phenotypes compared to Δ*MoMed15*, except that they exhibited higher resistance to IPT (see Fig. S6A in the supplementary material). It was found that the TGG codon (tryptophan) at position 607 of the *MoMed15* gene was substituted with TGA (terminator) (W607X) in 30-50 (Fig. 1C), and there was a T to C change at position 178 in the first intron of HR-1 (see Fig. S6B in the supplementary material).

To clarify the level of contribution of mutations in *MoMed15* to IPT resistance in 30-50 and HR-1 mutants, sequences of *MoMed15* mutations were complemented to Δ*MoMed15* transformant. As shown in Fig. S6C and D, Δ*MoMed15*-C^30-50^ and Δ*MoMed15*-C^HR-1^ displayed similar level of resistance to IPT as Δ*MoMed15*, while Δ*MoMed15*-C obtained similar level of IPT sensitivity as H08-1a, suggesting that the *MoMed15* mutations were only responsible for moderate IPT resistance in 30-50 and HR-1 mutants, while high IPT resistance should simultaneously cause by additional resistance genes.

**Fig 5:**
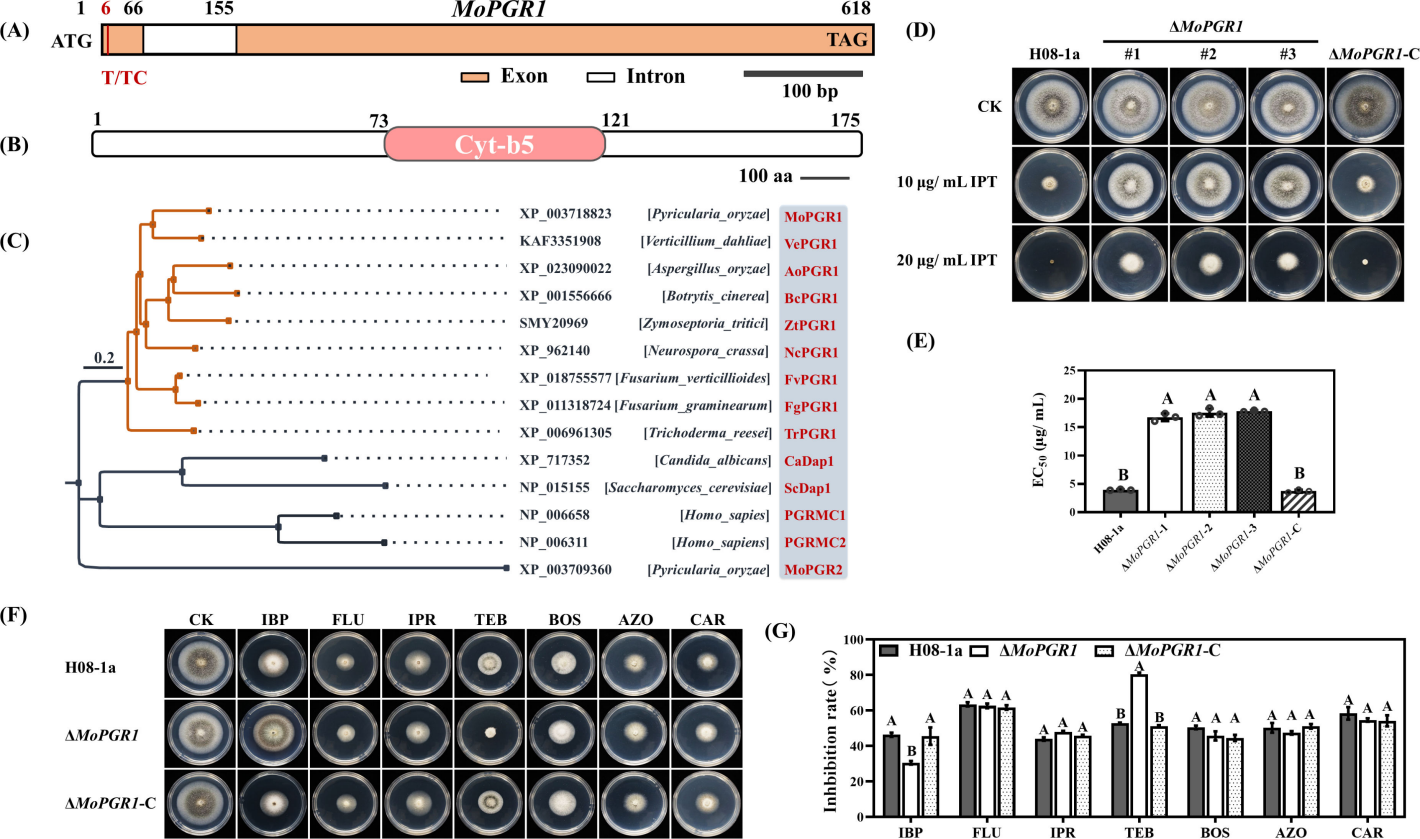
Double mutations in *MoPGR1* and *MoMed15* resulted in high levels of resistance to IPT in mutant 30-50. (**A**) *MoPGR1* gene has a shifted-code mutation (T to TC) in mutant 30-50. (**B**) The MoPGR1 amino acid sequence contains a conserved cytochrome b5-like heme/steroid binding structural domain identified using the NBCI protein database (https://www.ncbi.nlm.nih.gov/Structure/cdd/wrpsb.cgi). (**C**) Phylogenetic analysis of MoPGR1 homologous proteins in different species. Trees were constructed based on Mega 6.0 using the neighbor-joining method. (**D**) Δ*MoPGR1* transformants increased resistance to IPT, and Δ*MoPGR1*-C transformant restored the IPT sensitivity. A 3-mm mycelial plug of each strain was inoculated on PDA or PDA amended with 10, 20 µg/mL IPT and then incubated at 27°C for 5 days. (**E**) Statistical analysis of the IPT EC_50_ values of Δ*MoPGR1* and Δ*MoPGR1*-C transformants. The data presented are the mean ± SD (*n* = 3). Bars followed by the same letter are not significantly different according to a least significant difference test at *P* = 0.01. (**F**) Sensitivity of Δ*MoPGR1* transformants and Δ*MoPGR1*-C transformants to fungicides with different modes of action. H08-1a and transformants were inoculated on PDA or PDA amended with 30 µg/mL IBP, 4 µg/mL FLU, 20 µg/mL IPR, 0.3 µg/mL TEB, 30 µg/mL boscalid (BOS), 1 µg/mL azoxystrobin (AZO) or 0.3 µg/mL CAR, and then incubated at 27°C for 5 days. (**G**) Statistical analysis of mycelial inhibition of Δ*MoPGR1* transformants and Δ*MoPGR1*-C transformants to different fungicides.

Further analysis revealed that the *MoIRR* was missing the AG bases at positions 397 and 398 in the HR-1 mutant, and its sensitivity to IPT was similar to the Δ*MoMed15*Δ*MoIRR* double-knockout transformant, suggesting that co-mutations in the *MoIRR* and *MoMed15* genes were the reason for the high resistance in the HR-1 mutant (see Fig. S6B in the supplementary material). However, there were no *MoIRR* nucleotide sequence changes in 30-50 mutant. To investigate the molecular mechanism of high IPT resistance in the 30-50 mutant, SNP and InDel were extensively validated by whole-genome sequencing and gene knockout. It was found that *MGG_00274* had an insertion at position 6 (6 dup T), resulting in a frame-shift mutation (Fig. 5A). *MGG_00274* encoded a protein containing the cytochrome b5-like heme/steroid binding structural domain that was homologous to PGRMC1 in mammals and Dap1 in *S. cerevisiae*, so we named this protein as MoPGR1 (Fig. 5B and C).

To investigate whether MoPGR1 was involved in the regulation of sensitivity to IPT in *M. oryzae*, we constructed *MoPGR1* knockout, complemented and overexpression transformants by double-joint PCR and vector expression (see Fig. S7A and B in the supplementary material). The results showed that Δ*MoPGR1* knockouts significantly increased the resistance to IPT and that the Δ*MoPGR1*-C complementation restored the IPT sensitivity of Δ*MoPGR1* back to wild-type H08-1a (Fig. 5D and E), while the OE*MoPGR1* transformants were hypersensitive to IPT (see Fig. S7C and D in the supplementary material). Further phenotypic investigation showed that Δ*MoPGR1* transformants exhibited decreased sensitivity to IBP, a fungicide with the same mode of action as IPT, and increased sensitivity to DMI fungicide tebuconazole (Fig. 5F and G), explaining why the 30-50 mutant was more sensitive to DMIs than *MoMed15* mutants or Δ*MoMed15* transformants. These results indicated that *MoPGR1* serves as a novel gene conferring IPT sensitivity in *M. oryzae*.

MoPGR1 was also essential for virulence in *M. oryzae,* although there was no significant difference between Δ*MoPGR1* and wild-type H08-1a for growth and coping with environmental stress (see Fig. S8 in the supplementary material).

### MoIRR binds to the promoter of the *MoPGR1* gene

**Fig 6:**
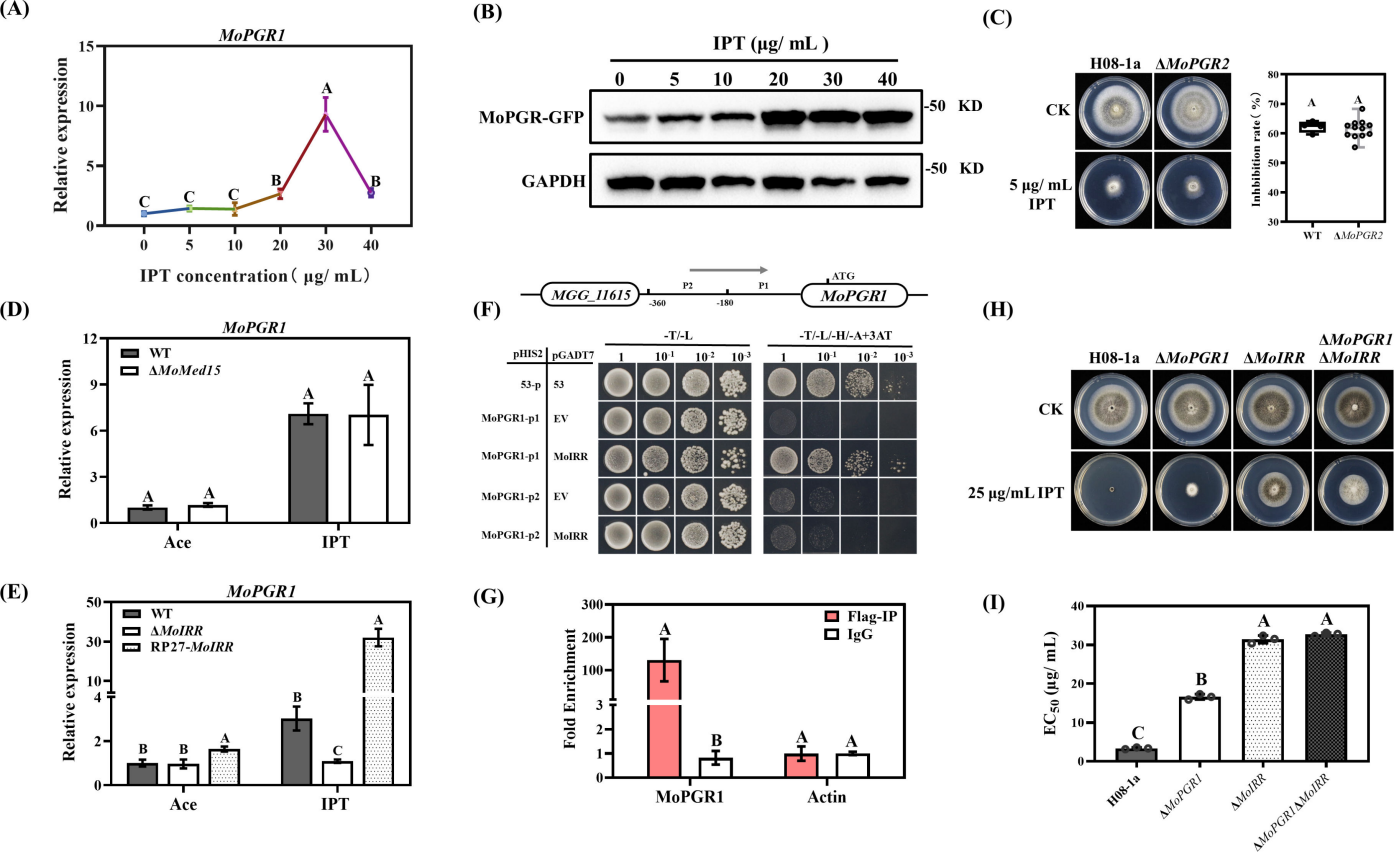
MoIRR activated the expression of MoPGR1 under IPT stress. (**A**) Expression of *MoPGR1* under different concentrations of IPT stress detected by RT-qPCR. The *MoActin* gene was used as the internal reference for normalization. Each strain was cultured in PDB for 48 h and then treated with 0, 5, 10, 20, 30, and 40 µg/mL IPT for 3 h, respectively. (**B**) Protein levels of *MoPGR1* under different concentrations of IPT stress were evaluated by Western blot. GAPDH was used as an internal reference for Western blot assay. (**C**) MoPGR2, a paralogous protein of MoPGR1, was not involved in the regulation of sensitivity to IPT in *M. oryzae*. (**D**) *MoPGR1* expression in Δ*MoMed15* transformant detected by RT-qPCR. Wild-type H08-1a and Δ*MoMed15* transformant were cultured in PDB for 48 h, and then treated with 0 or 15 µg/mL IPT for 3 h, respectively. (**E**) *MoPGR1* expression in Δ*MoIRR* and OE*MoIRR* transformants detected by RT-qPCR. Wild-type H08-1a, Δ*MoIRR,* and OE*MoIRR* transformants were cultured in PDB for 48 h, and then treated with 0 or 15 µg/mL IPT for 3 h, respectively. The *MoActin* gene was used as the internal reference for normalization. (**F**) Verification of MoIRR interaction with the MoPGR1 gene promoter by yeast one-hybrid. pHIS2-53 and pGADT7-53 were used as a positive control, and the usage concentration of 3 AT was 60 mMol/L. (**G**) Verification of MoIRR interaction with the MoPGR1 gene promoter by ChIP-qPCR. Mouse IgG was used as a negative control, *MoActin* was used as a non-specific target gene. MoIRR-Flag overexpression strain was incubated in PDB for 48 h, then treated with 15 µg/mL IPT for 3 h. (**H**) Sensitivity of Δ*MoPGR1*Δ*MoIRR* to IPT. A 3-mm mycelial plug of each strain was inoculated on PDA or PDA amended with 5, 10, 15, 25, and 35 µg/mL IPT and then incubated at 27°C for 5 days. (**I**) Statistical analysis of the EC_50_ values of Δ*MoPGR1*Δ*MoIRR* to IPT. The data presented are the mean ± SD (*n* = 3). Bars followed by the same letter are not significantly different according to a least significant difference test at *P* = 0.01.

To investigate the expression pattern of MoPGR1 in *M. oryzae* in response to IPT exposure, we generated a MoPGR1-GFP transformant expressing a fusion protein of GFP using the MoPGR1 promoter (0.6 k) and coding region. Expression of MoPGR1 was induced by high concentrations of IPT, suggesting that MoPGR1 can be rapidly activated under IPT stress (Fig. 6A and B). Further research found that MoPGR2, a homologous protein of MoPGR1 with 30% amino acid similarity, was not associated with IPT resistance (Fig. 6C). This is in contrast to previous studies showing that *PGRMC1* and *PGRMC2* had similar functions, suggesting that *MoPGR1* is irreplaceable for the regulation of IPT resistance.

To clarify the role of regulators that activate *MoPGR1* expression, we determined the expression levels of *MoPGR1* in Δ*MoMed15* and Δ*MoIRR* transformants by RT-qPCR. Surprisingly, *MoPGR1* was expressed only through *MoIRR* activation and *MoMed15* did not affect its expression (Fig. 6D and E). Meanwhile, Yeast one-hybrid demonstrated that the MoIRR protein could directly bind to the MoPGR1 promoter P1 (180 bp) (Fig. 6F). Furthermore, the interaction of MoIRR with the P1 promoter was demonstrated by ChIP-qPCR *in vivo* (Fig. 6G). Finally, the resistance of Δ*MoPGR1*Δ*MoIRR* to IPT did not increase compared to the resistance level of the Δ*MoIRR* transformant, indicating that MoIRR and MoPGR1 act together in the same resistance regulatory network (Fig. 6H and I). These results indicate that MoIRR can directly bind to the MoPGR1 promoter and activate its expression under IPT stress.

## DISCUSSION

Frequent use of site-specific fungicides for disease control can lead to the emergence and selection of resistant subpopulations and eventually control failure. Due to frequent exposure, it was going to be just a matter of time before IPT-resistant populations would emerge in Chinese rice fields. After all, IPT has been used frequently for rice blast management in China and East Asia since the 1970s (5). Unveiling the mechanism of resistance may lead to quicker detection methods and more effective strategies to avoid control failures in the future.

In our previous study, mutations in the transcription factor MoIRR were shown to play a key role in the development of moderate resistance to IPT in some strains of *M. oryzae* (15). Other laboratory-generated mutants also revealed similar phenotypes but did not possess the same molecular mechanism of resistance. Therefore, in this study, whole-genome sequencing was applied to further investigate the mechanism of IPT resistance in the resistant mutants without MoIRR mutations. Mutations in MoMed15 caused moderate resistance to IPT as confirmed through knockout and complementary transformation of the *MoMed15* gene. MoMed15 is considered a subunit of the transcriptional co-activation Mediator complex, homologous to GAL11 in *S. cerevisiae* and Med15 in *C. albicans*. The mutations in GAL11/Med15 enhance tolerance to acids, oxidation, and osmotic stress in *S. cerevisiae* (27). MoMed15 (syn MoCdtf1) was recently shown to be a key regulator for growth and development, colony pigmentation, conidia production, and appressorium formation, and was shown to be involved in the cAMP response pathway in concert with the transcription factor MoSom1 in *M. oryzae* (28). AfMed15 is also involved in the regulation of development, virulence, and autophagy in *A. fumigatus* (29).

Transcription activation regulates gene expression and is a common endpoint for many signaling pathways, including those that control cell growth and response to environmental stress (30). Previous studies have shown that some transcription activators recognize and bind specific DNA sequences on target promoters, then recruit the Mediator complex, which, in turn, recruits the Pol II complex to initiate transcription of the target gene (31). Med15 is a critical Mediator complex subunit that binds to transcription activators, mainly to the Zn_2_Cys_6_ family transcription factors in fungi. In mammals, the ARC105/Med15 KIX domain binds to the SREBP (sterol regulatory element-binding protein) and regulates cholesterol and fatty acid homeostasis (23). In *S. cerevisiae*, the Gal11/Med15 KIX domain can bind to the Zn_2_Cys_6_ transcription factors Oaf1, Gal4, and Pdr1 to promote the expression of fatty acid-dependent genes, galactose-dependent genes, and multidrug resistance genes, respectively (20, 22, 32). A follow-up study showed that the ABD region of Med15 also binds to the acidic activation domain of transcription factors (33, 34). TrGAL11 interacts with the Zn_2_Cys_6_ transcription factor Xyr1 to promote the recruitment of mediators and RNA Pol II to ensure the cellulase gene expression in *T. reesei* (21). Blocking the interaction between transcriptional activators and the Mediator subunit Med15 has been demonstrated to counteract fungicide resistance (35), but in this study, the loss of function of MoMed15 caused the development of resistance rather than increased sensitivity to IPT. Stimulating the expression of corresponding transcriptional activators, for example, MoIRR may therefore mitigate IPT resistance caused by the malfunction of MoMed15.

Analysis of sensitivity to fungicides indicated that strains sensitive to IPT were also sensitive to IBP, but resistant to TEB. Cross-resistance or in this case cross-sensitivity between fungicide of the same mode of action, such as IPT and IBP, is expected. Both fungicides belong to the family of phospholipid biosynthesis inhibitors (FRAC 6). In this study, strains resistant to IPT were sensitive to TEB and vice versa. This inverse sensitivity relationship between fungicides of different modes of action is not expected and warrants further investigation. Nevertheless, the inverse sensitivity between IPT and TEB implies the potential for the use of DMI fungicides, for example, TEB against IPT-resistant pathogen populations.

Here, we report for the first time that MoMed15 interacts with the Zn_2_Cys_6_ transcription factor MoIRR, which, in turn, regulates the expression of xenobiotic detoxification genes to cause IPT toxicity. In drug or pesticide metabolism, organisms use xenobiotic detoxification enzymes to modify xenobiotic compounds for detoxification, unexpectedly, it may also become to be toxification (36, 37). In our case, expression of xenobiotic detoxification genes appeared to be detrimental to the survival of *M. oryzae* under IPT stress, suggesting that some xenobiotic detoxification genes may exacerbate the toxicity of IPT as in the metabolism of aflatoxin in *A. flavus* (38). Based on RNA-seq and RT-qPCR analyses, MoMed15 and MoIRR regulated the expression of a variety of xenobiotic detoxification enzymes, such as cytochrome P450, hydrolases, and ABC transporter proteins. It is common for xenobiotic-metabolizing enzymes to convert inactive, non-toxic, or low-toxicity compounds into more active or more toxic products. In recent studies, the nematode cytochrome P450 enzyme CYP35C1 biologically activated selective imidazothiazole nematicide selectivin, into selectivin sulfoxide metabolite, which led to specific and efficient nematode killing (39). Identification of the key enzyme that increased the IPT toxicity will be the next focus of work.

**Fig 7:**
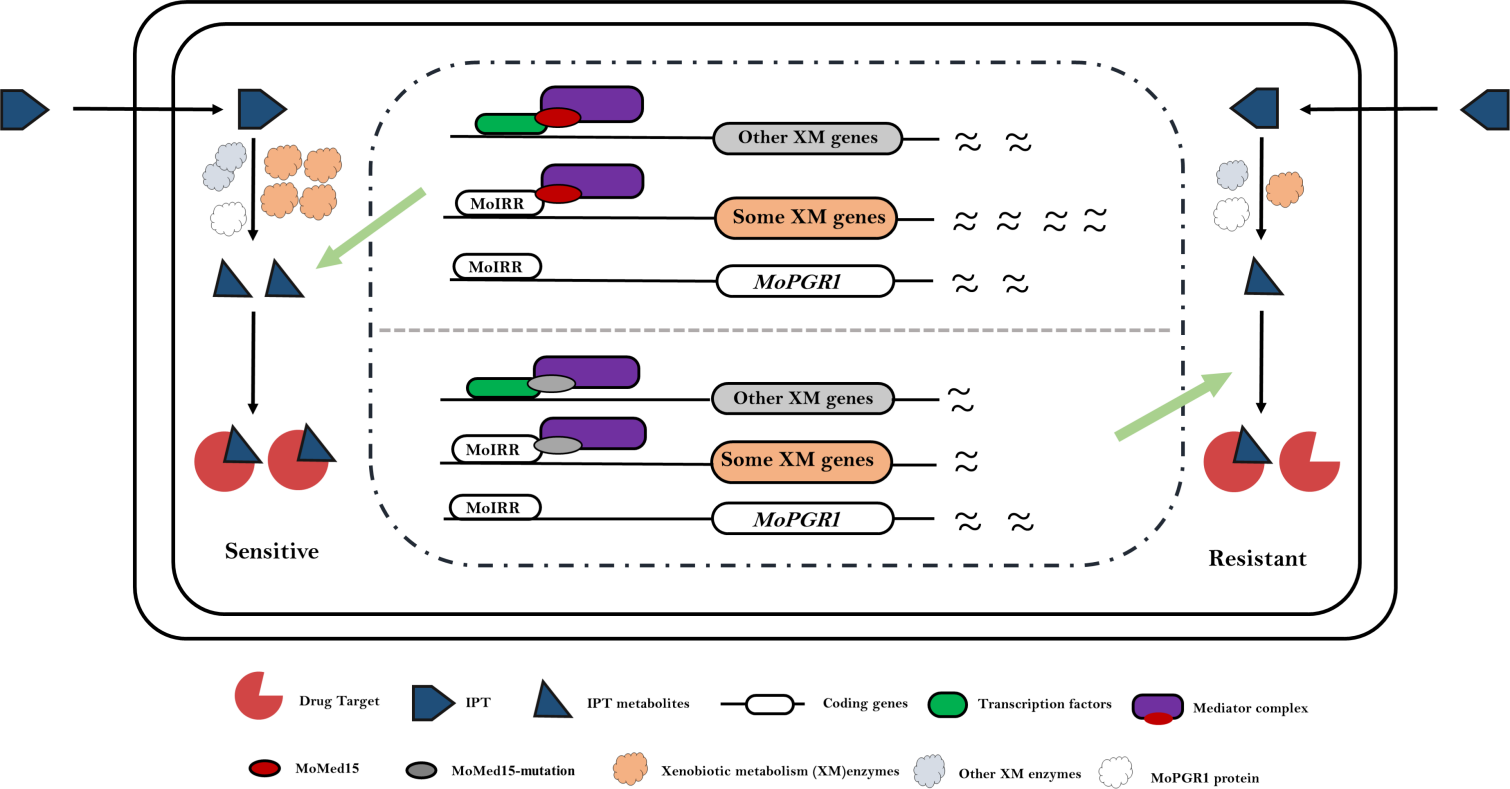
Model of the regulation of the IPT sensitivity mediated by MoMed15 in *M. oryzae*. Under the IPT stress, the Zn_2_Cys_6_ transcription factor MoIRR interacted with MoMed15 to recruit the Mediator complex, which strongly activated the expression of xenobiotic metabolism genes and thus enhanced the toxicity of IPT, whereas the mutation of MoMed15 resulted in the diminished expression of activated xenobiotic metabolism genes and toxicity of IPT, which led to the IPT resistance. In addition, MoIRR regulated the expression of MoPGR1 independently from MoMed15, showing a novel mechanism of resistance.

Our research revealed that *MoPGR1* is an important IPT-sensitive gene and that the MoIRR directly binds with its promotor. PGRMC1 or Dap1 is a heme-binding protein, often localized in the endoplasmic reticulum which activates cytochrome P450 enzymes and regulates the metabolism of drugs, hormones or lipids, and DNA damage responses (40–44). In *S. cerevisiae*, ScDap1 can directly interact with the target of azole drugs, ERG11, enhancing its enzyme activity, and thus the sensitivity of Δ*ScDap1* to azole drugs is significantly increased (40–42). The lack of *MoPGR1* also markedly enhanced the sensitivity to azole fungicide tebuconazole in *M. oryzae*. We believe that the absence of MoPGR1 may lead to a reduction in the methyltransferase activity of MoCYP51A and MoCYP51B1, the targets of the azole fungicides. Of greater significance is the way MoPGR1 may alter the IPT sensitivity in *M. oryzae*. We hypothesize that MoPGR1 may affect the activity of certain xenobiotic metabolizing enzymes such as cytochrome P450 or hydrolases, potentially being involved in the modification of the chemical structure of IPT. A potential model is that MoMed15 interacts with MoIRR and activates the expression of IPT bioactivating enzymes, while MoPGR1 activates the IPT bioactivating enzymes, which, in turn, leads to enhanced IPT toxicity (Fig. 7).

PGRMC1 or Dap1 is extensively involved in the metabolism of drugs, hormones, lipids, and DNA damage responses, but is less well studied for regulating the virulence of pathogenic fungi. In *Candida albicans*, CaDap1 influenced virulence through the regulation of chitin content within the cell wall (45). MoPGR1 has the potential to regulate the activity of critical metabolic enzymes, such as CYP51, which subsequently affect the concentrations of ergosterol and other cellular components. This modulation is significant in the context of the virulence in *M. oryzae*, and may be one of the reasons for the decreased virulence of Δ*MoPGR1* transformants.

The eukaryotic transcriptional cofactor complex SAGA, the Mediator complex, and chromosomal remodeling complexes, such as SWI/SNF play an important role in the regulation of global transcription (46). SAGA complex subunits Ada2 or Tra1 mediate resistance to DMIs and echinocandins in *C. albicans* (47, 48). SWI/SNF complex subunits, such as Snf2, Snf5, Swi3, and several other subunits, are involved in the DMI resistance in a variety of fungi (49–51). The deletion of MoSnf5, the core subunit of the SWI/SNF complex, significantly increased the IPT sensitivity, but did not alter the IBP sensitivity in *M. oryzae*, whereas deletion of the SAGA core subunit GCN5 did not affect IPT resistance (see Fig. S9A in the supplementary material), suggesting that MoIRR transcriptional activation of downstream target gene expression is dependent on the assistance of the SWI/SNF complex. The SWI/SNF complex was more important for MoPGR1 expression than the Mediator complex under IPT stress (Fig. 6D, see Fig. S9B in the supplementary material), suggesting that the SWI/SNF complex is involved in the MoPGR1 mediated IPT sensitivity.

In this study, we report a new mechanism conferring moderate resistance to IPT in *M. oryzae*. MoMed15 interacts with MoIRR to recruit the Mediator complex, which then activates the expression of xenobiotic metabolizing enzymes leading to IPT sensitivity. On the other hand, MoPGR1 activates the xenobiotic metabolizing enzymes, which, in turn, leads to enhanced IPT toxicity. When MoMed15 or MoPGR1 is mutated, the expression or activity of xenobiotic metabolizing enzymes is decreased, leading to IPT resistance (Fig. 7).

## MATERIALS AND METHODS

### Strains, media, and fungicides

The *M. oryzae* strains included wild-type isolate H08-1a, resistant mutants, and various transformants. The mutants 3-11, 5-7, 5-9, 30-31, and 30-50 were generated by gradual adaptation to IPT and described in a previous study (17). Detailed information of these mutants, transformants as well as a newly generated mutant HR-1 is presented in Table S1. All mutants are cultured on PDA (200 g potato, 20 g glucose, 20 g agar, add water to 1 L) medium for 5 days at 27°C in the dark. For vegetative growth, 3 mm × 3 mm mycelial plugs from the periphery of actively growing mycelium were transferred onto PDA or tomato oat agar (OTA, 150 mL tomato juice, 40 g oats, 0.6 g CaCO_3_, 20 g agar, add water to 1 L). The fungicide IPT (see Fig. S1A in the supplementary material) was dissolved in acetone to make an 8,000 µg a.i./mL stock solution. Sensitivity to IPT was assessed on PDA amended with IPT at 0, 1, 2, 5, 8, 15, 30, and 50 µg/mL. Growth inhibition was calculated, and regression against the logarithm of fungicide concentrations was analyzed to obtain EC_50_ values. Sensitivity to the phosphoro-thiolate fungicide iprobenfos (IBP), the sterol biosynthesis inhibitor tebuconazole (TEB), the phenylpyrrole fungicide fludioxonil (FLU), the dicarboximide fungicide iprodione (IPR), the TOR synthesis inhibitor rapamycin (RAP), and the methyl benzimidazole carbamate fungicide carbendazim (CAR) was assessed on PDA amended with corresponding fungicides at 20, 0.3, 4, 20, 0.15, and 0.3 µg/mL, respectively.

### Whole genome resequencing

Cetyltrimethylammonium bromide (CTAB) method was used to extract genomic DNA from the mutants 5-9 and 30-50. Genome sequencing was conducted on the Illumina HiSeq 4000 PE150 platform using 150 bp paired-end libraries with 500 bp inserts at Wuhan SeqHealth Technology Company. Lofreq (version 2.1.5) software was used to perform the SNP and InDel assays. To reduce unnecessary mutation sites, we specified strict screening criteria: (i) The mutation appears in the open reading frame region; (ii) the mutation can only appear once in a gene; (iii) the candidate gene is expressed in the hyphal growth stage; and (iv) The candidate gene is not mutated in the 1 a_mut genome because another moderate resistance type MR-2 (represented by 1 a_mut) has been confirmed to be caused by the mutations of MoIRR (17).

### Phylogenetic and 3D structural analysis of Med15 protein

The amino acid sequences of the MoMed15 and BLAST Servers at NCBI from the *Magnaporthe* genome (https://www.ncbi.nlm.nih.gov/genome/51706) were used. Phylogenetic trees were constructed by comparing the identified amino acid sequences using the neighbor-joining method (the number of bootstrap replications was 1,000) in MEGA7.0. 3D structure of the KIX domain of the Med15 protein was predicted by the website Robetta (https://robetta.bakerlab.org/).

### Genetic manipulations including knockout, complementation, and overexpression

As mutations were identified in *MoMed15* (*MGG_11346*), genetic transformation was carried out to validate the role of *MoMed15* in IPT resistance. Double-joint PCR was used to generate the knockout constructs of *M. oryzae MoMed15* (52). Three knockout transformants for each gene were obtained and confirmed, and one transformant was randomly selected from each group for further analysis. To generate complemented transformants of the Δ*MoMed15* knockout transformant, the complete *MoMed15* genomic region, including its upstream 2 kb region, was inserted into the plasmid pGTN for transformation. Δ*MoMed15*Δ*MoIRR* double knockout transformants were obtained with two resistance genes, that is, hygromycin and neomycin. Overexpression transformants were obtained through the construct including the MoMed15-coding region and the 500 bp promoter region of RP27 in the plasmid pKNRG. The genetic transformation was conducted using PEG-mediated protoplast transformation.

### Evaluation of stress sensitivity

To test sensitivity against different stresses, mycelial growth was assayed after incubation at 27°C for 5 days on PDA plates and PDA was amended with 120 g/L glycerol, 0.8 M NaCl, 1.2 M sorbitol, and 0.025% SDS (wt/vol), respectively.

### Analysis of fungicide cross-resistance

The sensitivity of *MoMed15* mutants to the fungicides iprobenfos, propiconazole, fludioxonil, iprodione, rapamycin, and carbendazim was determined. Graphs were generated by plotting the inhibition rates of fungicides against IPT and other agents on the horizontal and vertical axes, respectively. The potential presence of cross-resistance between these two categories of agents is examined through Spearman rank correlation analysis. Robust cross-resistance was identified when the statistical significance level (*P*) was less than 0.05 and the correlation coefficient (ρ) exceeded 0.6.

### RNA preparation and qRT-PCR

Mycelia from the relevant strains were collected under the specific conditions and times, frozen rapidly in liquid nitrogen, and stored at −80°C until use. Total RNA isolation was conducted using the TRIzol method. cDNA was prepared using a HifairII 1st Strand cDNA Synthesis kit (YEASEN Biotech Co., Ltd) with oligo (dT). RT-qPCR was performed with ChamQTM SYBR qPCR Master Mix (Vazyme biotech co., Lth) on a Bio-Rad CFX96 real-time PCR detection system. The comparative cycle threshold (CT) method was used for data analysis and the relative fold difference was expressed as 2^−ΔΔCT^ (53). As an internal reference, primers for *MoActin* were used for each quantitative real-time PCR analysis. Primer sequences are shown in Table S2.

### Yeast two-hybrid analysis

The full-length cDNA sequences of *MoMed15* and *MoIRR* were amplified to verify the potential interaction between MoMed15 and MoIRR using the Y2H assay. The cDNA of *MoMed15* was inserted into the *Eco*RI site of the pGBKT7 vector containing the GAL4-binding domain, and *MoIRR* cDNA was inserted into the *Eco*RI site of the pGADT7 vector containing the yeast GAL4 activation domain. The plasmid pairs of pGBKT7-MoMed15/pGADT7-MoIRR were co-transformed into the AH109 using the LiAc/Carry-DNA/PEG3350 transformation method (54). The plasmid pairs of pGADT7-T/pGBKT7-53 and pGADT7-T/pGBKT7-Lam served as the positive and negative controls, respectively. The interaction of protein structural domains is accomplished using a similar approach.

### Transcriptional activation analysis

To minimize the frequency of incorrect positive results in yeast two-hybrid assays, it is recommended to evaluate the potential self-activation of bait proteins before proceeding with the definitive confirmation of interactions in yeast two-hybrid studies. The complete and partial versions of BDMoMed15 were diluted individually and administered on SD/-Trp-His plates to evaluate the growth of yeast. AH109 with the BD empty vector was utilized as a negative control in the experiment.

### Yeast one-hybrid analysis

The full-length cDNA sequence of *MoIRR* and *MoPGR1* promoter sequences were amplified to verify the potential interaction between MoIRR and *MoPGR1* promoter using Y1H assay. The promoter sequence of *MoPGR1* was split into two parts, which were inserted into the *Eco*RI and *Spe*I site of the pHIS2 vector. Yeast transformation is performed in the same way as Y2H. 3AT is used at a concentration of 90 mM. The plasmid pairs of pHIS2-53/pGADT7-53 served as positive controls.

### RNA sequencing

RNA sequencing was conducted on the Illumina HiSeq 4000 PE150 platform using 150 bp paired-end libraries with 500 bp inserts at Wuhan SeqHealth Technology Company. Transcriptome data quality control was performed using fastp (version 0.23.0) and over 35 million high-quality reads per sample were achieved. The RPKM (Reads per Kilobase per Million Reads) value was used as a measure of gene expression, a gene was considered differentially expressed gene, when log_2_ [fold change (Δ*MoMed15,* Δ*MoIRR* or Δ*MoMed15*Δ*MoIRR* _RPKM/H08-1a_RPKM) ]>1 or < −1 and *P*-value < 0.05 (55). GO analyses were performed using the DAVID Bioinformatics Resources online website (https://david.ncifcrf.gov/home.jsp) (56)

### GST-pull down

The cDNA sequence encoding N-terminal 555 amino acids of MoMed15 was amplified and cloned into the pET-32a vector which generated the MoMed15-N-His protein. The full-length cDNA sequence of MoIRR was amplified and cloned into the pGEX4T-2 vector which generated the GST-MoIRR protein. The constructed vectors were transformed into *E. coli* DE3 and the expression of recombinant proteins was induced with 0.5 mM IPTG (isopropyl b-D-1-thiogalactopyranoside) and purified using GST beads (L20925, TransGen Biotech, Beijing). Cell lysate supernatants containing GST-MoIRR protein were incubated with GST beads for 12 h at 4°C. The beads were washed three times with phosphate-buffered saline (PBS) containing 1% Triton X-100. Cell lysate supernatants containing MoMed15-N-His were incubated with GST-MoIRR beads for 12 h at 4°C. The beads were washed three times. Beads were dissolved in SDS-loading buffer, boiled for 5 min, ice bath for 5 min, and the supernatant was detected by Western blot.

### Co-IP assay

GFP-MoIRR and MoMed15-Flag vectors containing full-length cDNA sequences were constructed and co-transformed into wild-type H08-1a strain. GFP empty vector and MoMed15-Flag vector were co-transformed into wild-type H08-1a strain as a negative control. Co-expressed transformants were analyzed by Western blot with the monoclonal anti-Flag (66008, Proteintech, Hubei, China) and anti-GFP (66002, Proteintech, Hubei, China). For the Co-IP assay, fresh transformant mycelium (200 mg) was ground in liquid nitrogen and suspended in 0.5 mL of cell lysis buffer (P0013, Beyotime Biotechnology, Shanghai, China) containing 2 mM phenylmethylsulfonylfluoride (PMSF) and 50 mM MG132. After vortexing and centrifugation, the supernatant was incubated with anti-Flag Affinity Gel (P2282, Beyotime, Shanghai, China) at 4°C overnight. Proteins eluted from the anti-Flag Affinity Gel were analyzed by Western blot with the monoclonal anti-GFP antibody.

### Luciferase complementation assay

Full-length cDNA sequences of MoMed15 and MoIRR were constructed into nLUC and cLUC vectors, respectively, and the recombinant vectors were transformed into Agrobacterium GV3101. Agrobacterium containing nLUC-MoMed15 and cLuc-MoIRR was mixed 1:1 and infiltrated into tobacco leaves. Two days later, the tobacco leaves were observed by plant live imaging (NightSHADE L985, Berthold, Germany).

### Statistical analysis

Statistical differences were evaluated by one-way ANOVA with Duncan’s Multiple Range test in SPSS Version 19.0. Graphs were produced using GraphPad Prism 8. Correlations between fungicide sensitivity were analyzed with the Spearman correlation test in SPSS Version 19.0.

## Data Availability

The whole-genome sequencing data of 5-9 and 30-50 mutants have been deposited in the public archive of NCBI GenBank BioProject under accession number PRJNA1148345, SRA data: SRX25710100, SRX25710100. The RNA-Seq data of MoMed15 and MoIRR knockout mutants have been deposited in the public archive of NCBI GenBank BioProject under accession number PRJNA1149166, SRA data: SRX25751980–SRX25751997.

## SUPPLEMENTAL MATERIAL

Supplemental Tables and Figures mbio.01778-24-s0001.pdf Tables S1-S3; Figures S1-S9.

ASM does not own the copyrights to Supplemental Material that may be linked to, or accessed through, an article. The authors have granted ASM a non-exclusive, world-wide license to publish the Supplemental Material files. Please contact the corresponding author directly for reuse.
