# Supplementary material for: The Mediator complex subunit MoMed15 plays an important role in conferring sensitivity to isoprothiolane by modulating xenobiotic metabolism in M. oryzae
Source: mBio. 2024 Nov 12;15(12):e01778-24. doi: 10.1128/mbio.01778-24 (PMC11633134; doi:10.1128/mbio.01778-24)
Supplement: Supplemental Tables and Figures — Tables S1-S3; Figures S1-S9. [file mbio.01778-24-s0001.pdf]

Table S1. *Magnaporthe oryzae* strains used in this study

Strains	Description	Reference
H08-1a	Wild type strain	Hu et al., 2014
3-11	MoMed15 point mutant strain	Meng et al., 2023
5-7	MoMed15 point mutant strain	Meng et al., 2023
5-9	MoMed15 point mutant strain	Meng et al., 2023
30-31	MoMed15 point mutant strain	Meng et al., 2023
30-50	MoMed15 and MoPGR1 mutant strain	Meng et al., 2023
HR-1	MoMed15 and MoIRR mutant strain	This study
Δ*MoIRR*	*MoIRR* (*MGG_04843*) knockout transformant	Meng et al., 2023
OE*MoIRR*	The RP27 500 bp promoter with the *MoIRR* CDs	Wang et al., 2022
Δ*MoMed15*	*MoMed15* (*MGG_11346*) knockout transformant	This study
Δ*MoMed15*-C	*MoMed15* complemented transformant with 2k native promoter and full length of *MoMed15* in the background of Δ*MoMed15*	This study
OE*MoMed15*	The RP27 500 bp promoter with the *MoMed15* CDs	This study
Δ*MoMed15*Δ*MoIRR*	*MoMed15* and *MoIRR* double knockout transformant	This study
Δ*MoMed15*-C-OE*MoIRR*	The RP27 500 bp promoter with the *MoIRR* CDs in the background of Δ*MoMed15*	This study
Δ*MoIRR*-C-OE*Med15*	The RP27 500 bp promoter with the *MoMed15* CDs in the background of Δ*MoIRR*	This study
Δ*MoIRR*-C$^{30\text{-}50}$	*MoMed15* of 30-50 mutant with 2k native promoter and full length CDs in the background of Δ*MoIRR*	This study
Δ*MoIRR*-C$^{HR\text{-}1}$	*MoMed15* of HR-1 mutant with 2k native promoter and full length CDs in the background of Δ*MoIRR*	This study
Δ*MoPGR1*	*MoPGR1* (*MGG_00274*) knockout transformant	This study
Δ*MoPGR1*-C	*MoPGR1* complemented transformant with 600 bp native promoter and full length of *MoPGR1* gene in the background of Δ*MoPGR1*	This study
OE*MoPGR1*	The 1500 bp H3 promoter with the *MoPGR1* CDs	This study
Δ*MoPGR2*	*MoPGR2* (*MGG_06654*) knockout transformant	This study
Δ*MoPGR1*Δ*MoIRR*	*MoPGR1* and *MoIRR* double knockout transformant	This study
Δ*MoGcn5*	*MoGcn5* (*MGG_03677*) knockout transformant	This study
Δ*MoSnf5*	*MoSnf5* (*MGG_06315*) knockout transformant	This study

Table S2. Primers used in this study

Name	Sequence (5′-3′)	Primer description
MoIRR-Check-F	TTTCTTTGCCAGTGAGAACCC	Primers for *MoIRR* amplification
MoIRR-Check-R	GGTAGCGTTAAACGTTTCCA	
DJ1-MoMed15-5F	CGACCTGTTGTTATAAACCCAT	*MoMed15* knockout primers
DJ1-MoMed15-5R	TCAATATCATCTTCTGTCGAGCGAGTGTCTGAGATGACTTT	
DJ1-MoMed15-3F	AGATGCCGGATCCACTTAACGGAGAATATTTTCCCAGCAGTT	
DJ1-MoMed15-3R	CTTAGTCATGACCCCTGTCA	
DJ3-MoMed15-F	GTTACGTACCACTAGCTAGCT	
DJ3-MoMed15-R	TACCCCCTCATTCGTCACAC	
MoMed15-Check-F	ATGTTACGTACCACTAGCTAGCT	Validation primers for *MoMed15* knockout transformants
MoMed15-Check-R	CCGTTCGCTTCCAAGATGTAGTT	
DJ1-MoMed15/MoIRR-3F	GCCTTCTTGACGAGTTCTTCTGAGGAGAATATTTTCCCAGCAGTT	Primers for *MoMed15* knockout in Δ*MoIRR* transformant
DJ1-MoMed15/MoIRR-5R	GGTTCACGTAGTGGGCCATCGCGAGTGTCTGAGATGACTTT	
PGTN-MoMed15-F	CCCCTCGAGGTCGACGGTATCGATGTTACGTACCACTAGCTAGCT	Primers for *MoMed15* amplification in complemented transformants
PGTN-MoMed15-R	CCAGCACCTCTAGAACTAGTGGATCCGTTCGCTTCCAAGATGTAGTT	
PTNRG-MoMed15-F	CCCAATCTTCAAACCTGCAGATGGCTGGCAATATGCCTCAGA	Primers for *MoMed15* amplification in overexpression transformants
pTNRG-MoMed15-R	CCAGCACCAGCACCTCTAGAGTTCGCTTCCAAGATGTAGT	
BD-MoMed15-F	CATATGGCCATGGAGGCCATGGCTGGCAATATGCCTCAGA	Amplification primers of *MoMed15* for constructing it into pGBKT7 vector
BD-MoMed15-R	CAGGTCGACGGATCCCCGGTCAGTTCGCTTCCAAGATGTAG	
AD-MoMed15-F	CCATGGAGGCCAGTGAATTCATGGCTGGCAATATGCCTCAGA	Amplification primers of *MoMed15* for constructing it into pGADT7 vector
AD-MoMed15-R	GCTCGAGCTCGATGGATCCCTCAGTTCGCTTCCAAGATGTAG	
BD-ABD-F	CATATGGCCATGGAGGCCATGCACATGCAGAACAATGTCAACC	Amplification primers of *MoMed15* encoding different domains for constructing them into pGBKT7 vector
BD-ABD-R	CAGGTCGACGGATCCCCGGCTACCCAACGCCGGGTTCGGG	
BD-Med15-C-F	CATATGGCCATGGAGGCCATGCTCCAGGCGAACCAACAAG	

BD-GAL11-F	CATATGGCCATGGAGGCCATGCCAGGCCCCCAGATG	
BD-GAL11-R	CAGGTCGACGGATCCCCGGCTACGGCTGCTGTCCCTGCTG	
qRT-MoMed15-F	AAAACCAGGCTATCAACCA	Primers for evaluating *MoMed15* expression
qRT-MoMed15-R	GTGCGAGTCCATTTACCG	
qRT-MGG_02982-F	GCTCCTTTGGAACTTTGACA	Primers for evaluating *MGG_02982* expression
qRT-MGG_02982-R	TCCTCGGCTTGAACTTGAC	
qRT-MGG_01391-F	CCCAGAACCAGTTCGTCAG	Primers for evaluating *MGG_01391* expression
qRT-MGG_01391-R	GCCAGCCAGCTTCACATC	
qRT-MGG_08498-F	GACCCGCAGGGACAAGAA	Primers for evaluating *MGG_08498* expression
qRT-MGG_08498-R	GCACAAGAGGTAGGTGGAAAA	
qRT-MGG_01425-F	GTTATGCCGCTGGACCTT	Primers for evaluating *MGG_01425* expression
qRT-MGG_01425-R	AGGAGCACAGCCTGGAAA	
qRT-MGG_03070-F	CCTTTGGGTTTGCTCTGC	Primers for evaluating *MGG_03070* expression
qRT-MGG_03070-R	ATGTATTCGTCGGTCGTGTAG	
qRT-MGG_07848-F	GGCACGCCGCTACACTA	Primers for evaluating *MGG_07848* expression
qRT-MGG_07848-R	CACTTGCGAGCATTGAACT	
qRT-MGG_09677-F	CCAAGCTGAGCAGGGACGAG	Primers for evaluating *MGG_09677* expression
qRT-MGG_09677-R	GCCCGCACGCTGGAAAT	
qRT-MGG_02329-F	TATGATGCGTGAGAACAGGG	Primers for evaluating *MGG_02329* expression
qRT-MGG_02329-R	GCAGGGTAAAGAGGTAGGAAA	
qRT-MGG_05826-F	TACCGCAACTTTGACAAGAACA	Primers for evaluating *MGG_05826* expression
qRT-MGG_05826-R	TCGTCGCACAGCTCCCT	
qRT-MGG_03793-F	GCCATTTCCCGACAGAC	Primers for evaluating *MGG_03793* expression
qRT-MGG_03793-R	GTCGTAGAAGATGTAGCCCTC	
qRT-MGG_06069-F	AAGGACCCTGTGCCGTTTA	Primers for evaluating *MGG_06069* expression
qRT-MGG_06069-R	CCGTTGTTGGCAATGAGC	
qRT-MGG_10046-F	CTGACTGGGTTTCTGGAGG	Primers for evaluating *MGG_10046*

qRT-MGG_10046-R	ATGGAGCCGAGGGAGTT	expression
qRT-Actin-F	TCTTCGAGACCTTCAACGCC	Primers for evaluating *MoActin* expression
qRT-Actin-R	ACCGGAGTCGAGCACGATAC	
DJ1-MoPGR1-5F	ATCACACCTGTCAATTGCCA	*MoPGR1* knockout primers
DJ1-MoPGR1-5R	TCAATATCATCTTCTGTCGATGGAAAGGCGTCTTGGTCGA	
DJ1-MoPGR1-3F	AGATGCCGGATCCACTTAACCGTGTGGTTCTGATGGTTCA	
DJ1-MoPGR1-3R	TGTCCAAGCTCGTTAGATTGG	
DJ3-MoPGR1-F	CACACCAAACGATGAAGAGC	
DJ3-MoPGR1-R	ATTGGCTACATGCTGATGCA	
MoPGR1-Check-F	ATGGATTACGTTGAGCAGCG	Validation primers for *MoPGR1* knockout transformants
MoPGR1-Check-R	TTTCTTCTCTTCATCACCGAC	
PTNHF-MoPGR1-F	ACCCAATCTTCAAACCCGGGATGGATTACGTTGAGCAGCG	Amplification of *MoPGR1* in overexpression transformants
PTNHF-MoPGR1-R	TCGACGGTATCGATAAGCTTTTTCTTCTCTTCATCACCGAC	
PGTN-MoPGR1-F	CCCCTCGAGGTCGACGGTATCGATATCACACCTGTCAATTGCCA	Amplification of *MoPGR1* in complemented transformants
PGTN-MoPGR1-R	CCAGCACCTCTAGAACTAGTGGATCCTTTCTTCTCTTCATCACCGAC	
qPCR-MoPGR1-F	AAAACTTTGCAGGCAGGGAC	Primers for evaluating *MoPGR1* expression
qPCR-MoPGR1-R	CTCAGGGCAGCCAACTCAT	
DJ1-MoPGR2-5F	CGACTTCTATCGACCGTTGT	*MoPGR2* knockout primers
DJ1-MoPGR2-5R	TCAATATCATCTTCTGTCGACGGTGCGAGTCTAGACAATG	
DJ1-MoPGR2-3F	AGATGCCGGATCCACTTAACCTGCGTATCGTCAAAAATCT	
DJ1-MoPGR2-3R	TGGGGTGGTCAATAAAAGCC	
DJ3-MoPGR2-F	TTCTACTGTTAGCCGCCGGA	
DJ3-MoPGR2-R	GGCCCTATTCTGTAGAGTC	
MoPGR2-Check-F	ATGGCGGACGAAGGAGAATT	Validation primers for *MoPGR2* knockout transformants
MoPGR2-Check-R	TCAACCTATTGCCCGCCC	
DJ1-Gcn5-5F	ACAGGTGTGTTCTCGTATTC	*MoGcn5* knockout primers
DJ1-Gcn5-5R	TCAATATCATCTTCTGTCGACTGTGTCGTAGAGACCTCTA	

DJ1-Gcn5-3F	AGATGCCGGATCCACTTAACAAACGGTCGTTGGTTCAATT	
DJ1-Gcn5-3R	ATGAAGGTACTCCCGGCAGA	
DJ3-Gcn5-F	TAAGGTTACGGATAGTGCTCCG	
DJ3-Gcn5-R	TCACGAACCTAACGCAAAGG	
Gcn5-Check-F	ATGTCTACAGCAACAGAAGATA	Validation primers for *MoSnf5* knockout transformants
Gcn5-Check-R	TCATGGTTCAAGATGTGACCAC	
DJ1-Snf5-5F	AACAGAGCATGGCTGATGGA	
DJ1-Snf5-5R	TCAATATCATCTTCTGTCGAGATTATCTAGAGGCGCGCTC	
DJ1-Snf5-3F	AGATGCCGGATCCACTTAACTTGCACAACCAGATACTCGC	*MoSnf5* knockout primers
DJ1-Snf5-3R	AGAAGCCGTACCGCAAAAGA	
DJ3-Snf5-F	TAGGTGGCCTCTTGGGAATC	
DJ3-Snf5-R	AGAAACGCAGATGGTCCCT	
Snf5-Check-F	ATGGCAACCTCGGATCCC	Validation primers for *MoSnf5* knockout transformants
Snf5-Check-R	CAGGTGATTGAGAGGCCGAT	

Table S3. Potential resistance-associated SNP and Indel loci in the mutants 30-50 and 5-9

Chr	Position	Ref	Alt	H08_1a	30-50	5-9	Func.refGene	Gene.refGene	Gene description	Interpro Description
1	237238	T	C	0	0	1	exonic	*MGG_02014*	Putative uncharacterized protein [Source:UniProtKB/TrEMBL;Acc: G4MMR7]	
1	827343	C	T	0	0	1	intronic	*MGG_16049*	Putative uncharacterized protein [Source:UniProtKB/TrEMBL;Acc: G4MP73]	
1	5155013	C	G	0	0	1	exonic	*MGG_05419*	Putative uncharacterized protein [Source:UniProtKB/TrEMBL;Acc: G4MLD7]	
6	503506	G	T	0	0	1	exonic	*MGG_15370*	Metalloproteinase [Source:UniProtKB/TrEMBL;Acc: G4NFY3]	Metallopeptidase, catalytic domain superfamily
1	73929	T	C	0	0	1	exonic	*MGG_15986*	Putative uncharacterized protein [Source:UniProtKB/TrEMBL;Acc: G4MMA4]	
1	2877659	T	C	0	0	1	exonic	*MGG_14569*	Putative uncharacterized protein [Source:UniProtKB/TrEMBL;Acc: G4MM35]	Chromo/chromo shadow domain
2	1570504	G	A	0	0	1	exonic	*MGG_11346*	Putative uncharacterized protein [Source:UniProtKB/TrEMBL;Acc: G4MU85]	Gal11, coactivator domain
7	2801171	T	G	0	0	1	exonic	*MGG_14014*	Ubiquitin homeostasis protein lub1 [Source:UniProtKB/TrEMBL;Acc: G5EH30]	WD40 repeat
2	1570872	C	T	0	0.99	0	exonic	*MGG_11346*	Putative uncharacterized protein [Source:UniProtKB/TrEMBL;Acc: G4MU85]	Gal11, coactivator domain
3	6319429	C	T	0	1	0	exonic	*MGG_16972*	Putative uncharacterized protein [Source:UniProtKB/TrEMBL;Acc: G4N2T4]	Zinc finger, CCHC-type
5	3579549	A	T	0	1	0	exonic	*MGG_00233*	Fatty acid synthase S-	Acyl transferase domain

									acetyltransferase [Source:UniProtKB/TrEMBL;Acc: G4NDK2]	
5	4472002	A	G	0	1	0	exonic	*MGG_17555*	Putative uncharacterized protein [Source:UniProtKB/TrEMBL;Acc: G4MXX7]	
7	9643	G	A	0	0.97	0	exonic	*MGG_17846*	Putative uncharacterized protein [Source:UniProtKB/TrEMBL;Acc: G4NJP9]	Helicase, C-terminal
5	3451421	-	C	0	0.94	0	exonic	*MGG_00274*	Membrane-associated progesterone receptor component 1 [Source:UniProtKB/TrEMBL;Acc: G4NDA1]	Cytochrome b5-like heme/steroid binding domain

Note: Chr, Ref, and Alt represent chromosomes, sequences corresponding to reference genomes, mutant sequences corresponding to the sample genomes, respectively.

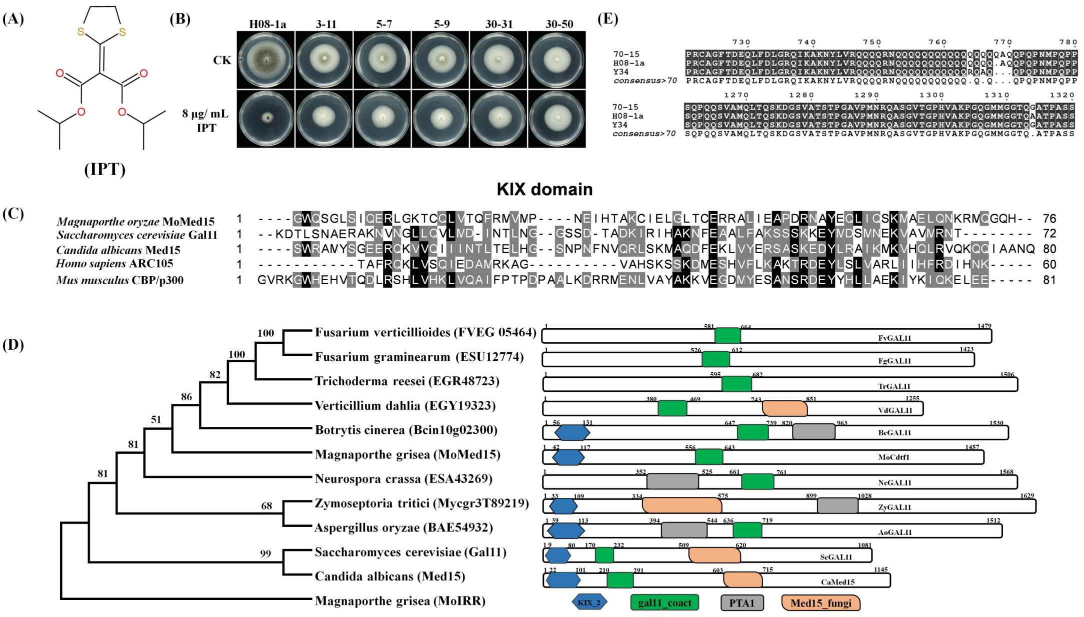

FIG S1 MoMed15 amino acid sequence and phylogenetic analysis. (A) Chemical structure of IPT. (B) Phenotypic analyses of some moderate resistance mutants for IPT resistance in a IPT resistance mutant library. (C) Alignment of amino acid sequence of KIX structural domain of MoMed15 in *Magnaporthe oryzae*, Gal11 in *Saccharomyces cerevisiae*, Med15 in *Candida albicans*, ARC15/Med15 in *Homo sapinens* and CBP/p300 in *Mus musculus*. (D) Phylogenetic analysis was performed by using amino acid sequences of MoMed15 homologous proteins in the NCBI database. (E) Differential analysis of amino acids of MoMed15 in different strains of *M. oryzae*.

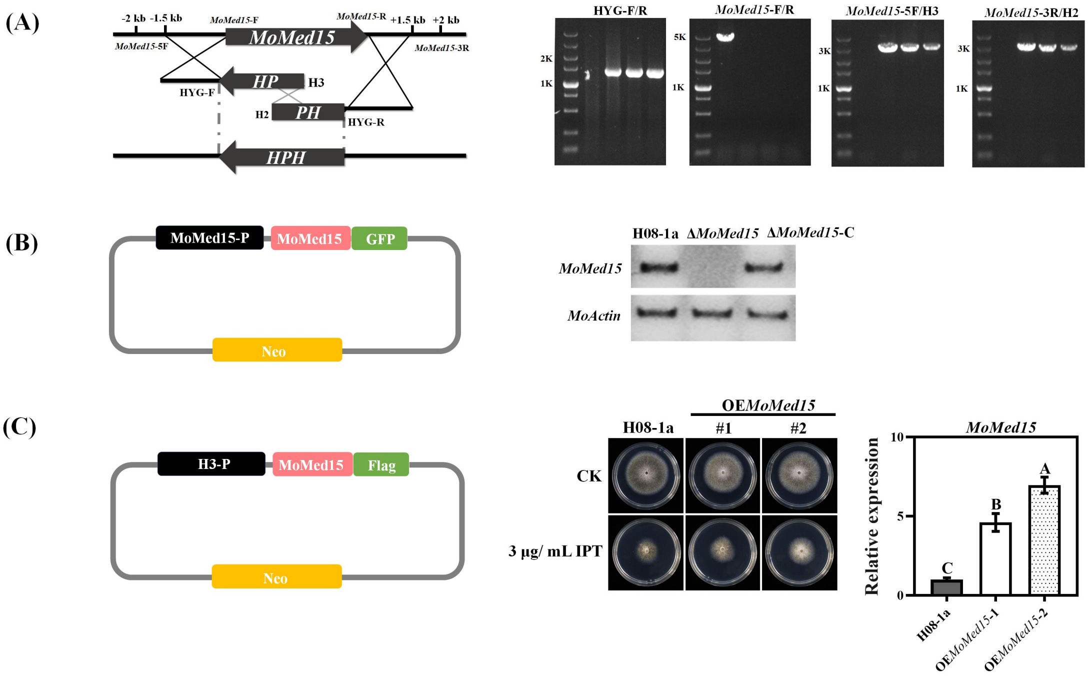

FIG S2 Validation of *MoMed15* gene knockout, complementation and overexpression. (A) Knockout strategy and verification of knockout transformants by primer-specific PCR. (B) Complementary vector and verification of complemented transformants by RT-PCR. (C) Determination of sensitivity of *MoMed15* overexpression transformants to IPT and detection of *MoMed15* expression in OE*MoMed15* transformants by RT-qPCR. The *MoActin* gene was used as the internal reference for normalization. Data presented are the mean ± SD (n = 3).

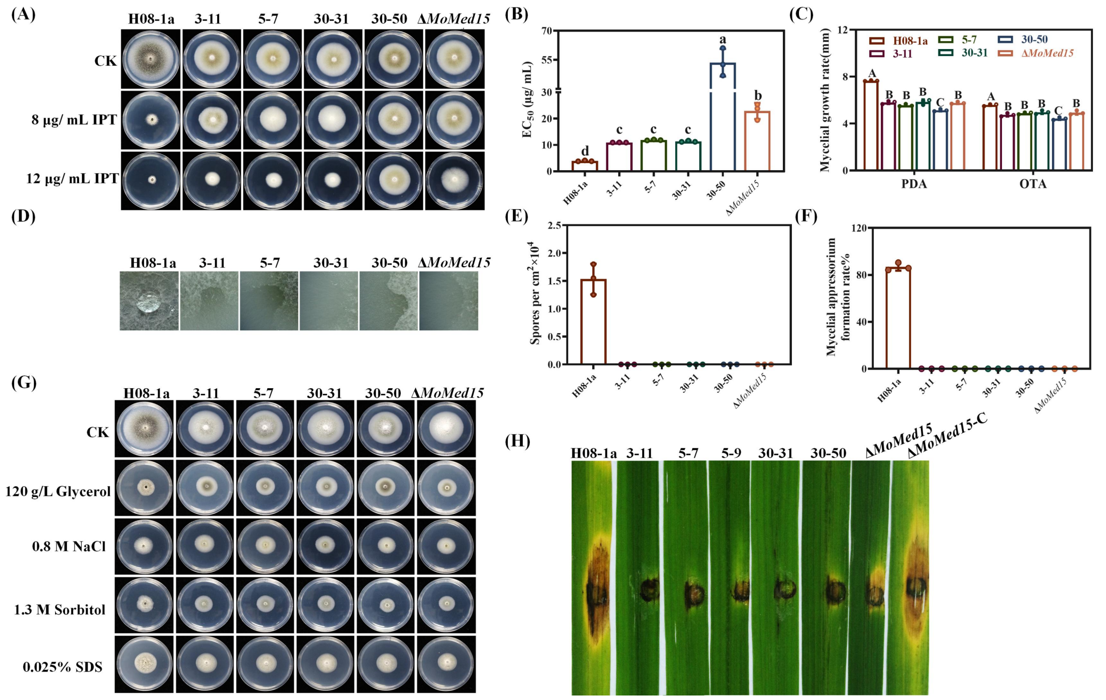

FIG S3 Determination of the fitness of Δ*MoMed15* transformants and *MoMed15* mutants. (A) Determination of sensitivity of Δ*MoMed15* transformants and *MoMed15* mutants to IPT. (B) Statistical analysis of the of Δ*MoMed15* transformants and *MoMed15* mutants to IPT. (C), (D), (E), (F), (G), and (H), Determination of growth rate, hydrophobicity, spore production, appressorium formation rate, sensitivity to glycerol, NaCl, sorbitol, and SDS, and virulence of Δ*MoMed15* transformants and *MoMed15* mutants, respectively.

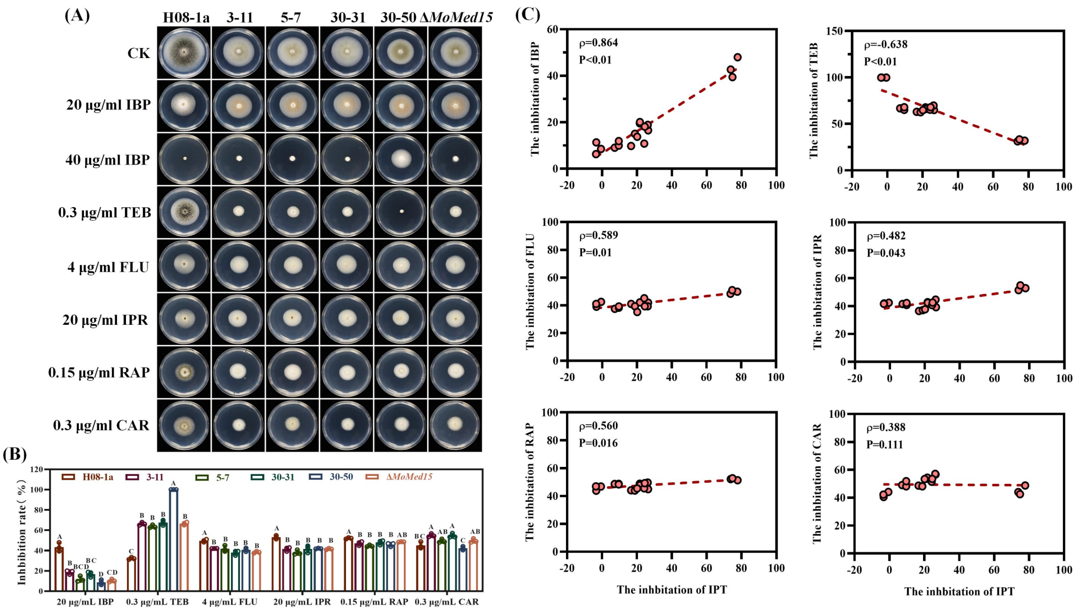

FIG S4 IPT showed positive cross-resistance with IBP, and negative cross-resistance with TEB. (A) Sensitivity of Δ*MoMed15* transformants and *MoMed15* mutants to fungicides with different modes of action. H08-1a and IPT resistant strains were inoculated on PDA or PDA amended with 20 or 40 μg/mL iprobenfos (IBP), 0.3 μg/mL tebuconazole (TEB), 4 μg/mL fludioxonil (FLU), 20 μg/mL iprodione (IPR), 0.15 μg/mL rapamycin (RAP), 0.3 μg/mL carbendazim (CAR), and then incubated at 27℃ for 5 days. (B) Statistical analysis of mycelial inhibition of Δ*MoMed15* transformant and *MoMed15* mutants to different fungicides. (C) Spearman correlation tests for cross-resistance between IPT with IBP, TEB, FLU, IPR, RAP, CAR. ρ=Spearman`s rho, p=p-value. Each point in the graph represents the inhibition rate of *MoMed15* mutants to different fungicides.

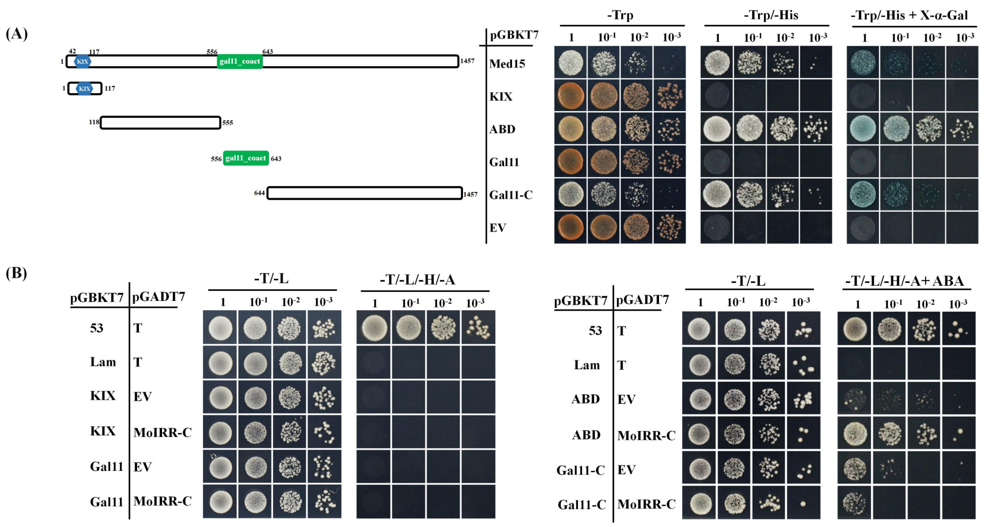

FIG S5 Demonstration of interaction between C-terminal of MoIRR and KIX-GAL11 region of MoMed15 by yeast two-hybrid assay. (A) The ABD and Gal11-C segment of MoMed15 exhibit transcriptional activity. KIX (1-117 aa), ABD (118-555 aa), Gal11 (556-643 aa), Gal11-C (644-1457 aa). AH109 with the BD empty vector was used as a negative control in the experiment. (B) Investigation of interaction between C-terminus of MoIRR and four MoMed15 segments by yeast two-hybrid assay. The utilization of 75 μg/ml Aureobasidin A (ABA) was necessary to inhibit the self-activation observed in both the complete sequence and specific structural regions of MoMed15 within yeast cells.

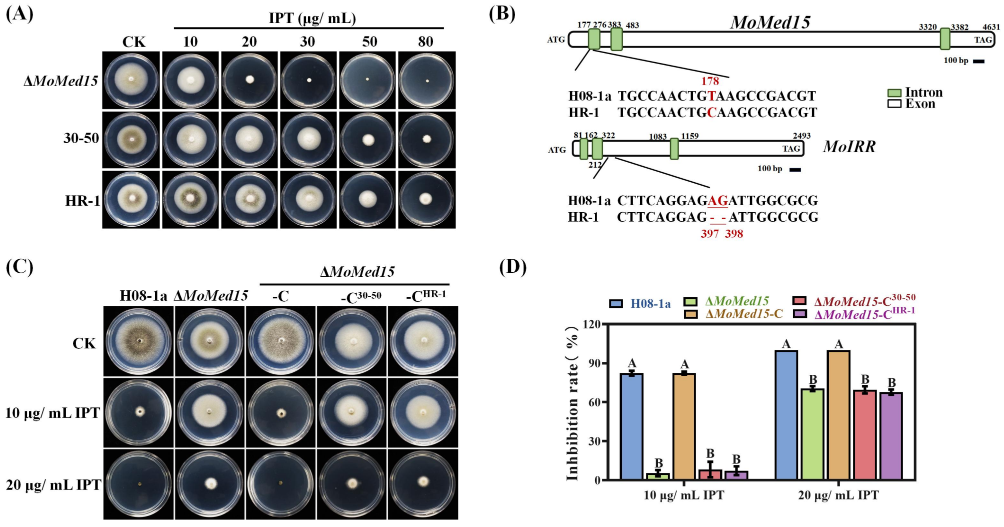

FIG S6 Effect of mutations of *MoMed15* to IPT resistance. (A) 30-50 and HR-1 mutants highly resistant to IPT. (B) *MoMed15* and *MoIRR* gene sequence changes in HR-1 mutants. (C) *MoMed15* single base mutant transformants showed moderate resistance to IPT. (D) Statistical analysis of mycelial inhibition of *MoMed15* single-base mutant transformants to IPT. Data presented are the mean ± SD (n = 3). Bars followed by the same letter are not significantly different according to a LSD test at P =0.01.

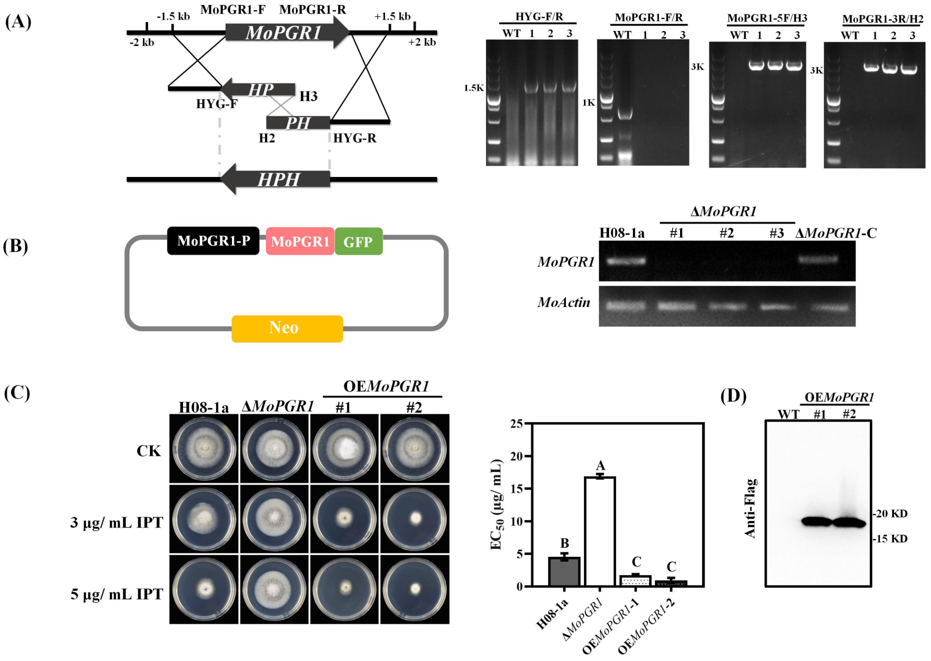

FIG S7 Knockout, complementation and overexpression of the *MoPGR1* gene. (A) Knockout and verification of *MoPGR1* knockout transformants. (B) Complementation and verification of *MoPGR1* complemented transformants. (C) OE*MoPGR1* transformants increased the IPT sensitivity. (D) Verification of *MoPGR1* overexpression transformants by Western blot.

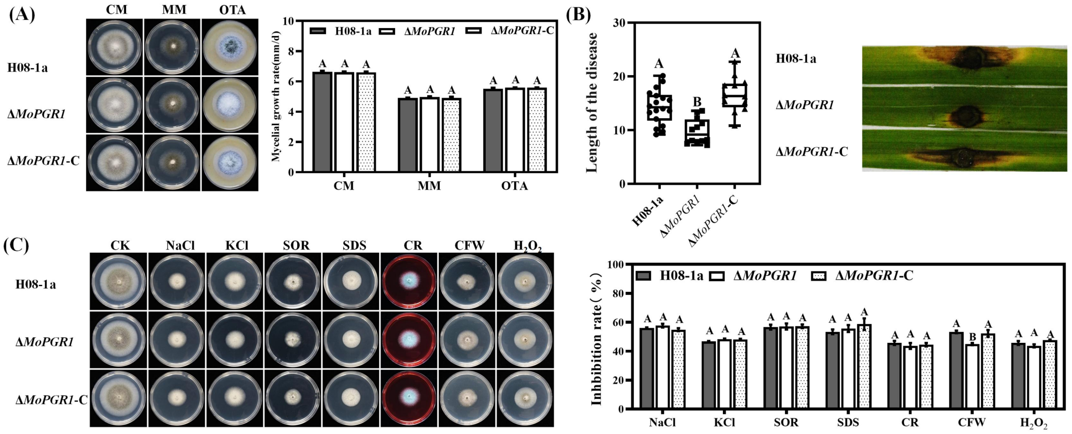

FIG S8 Environmental fitness of *MoPGR1* knockout and complemented transformants. (A) MoPGR1 did not influence the growth of *M. oryzae*. (B) MoPGR1 was required for virulence in *M. oryzae*. The Δ*MoPGR1* and Δ*MoPGR1*-C transformants were inoculated on rice leaves of ZH11 for 10 days. (C) MoPGR1 was not involved in the regulation of sensitivity to environmental stresses in *M. oryzae*.

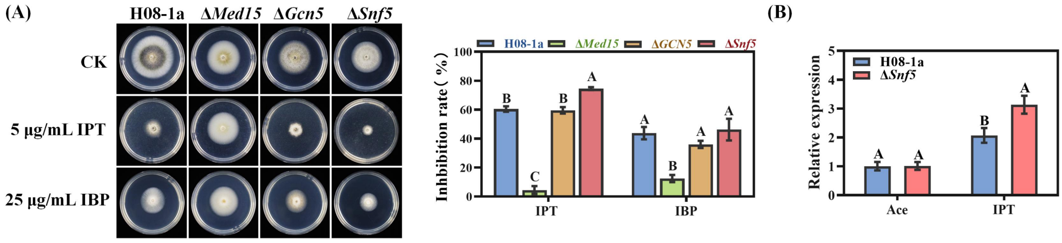

FIG S9 Deletion of *MoSnf5* increased the IPT sensitivity in *M. oryzae*. (A) Sensitivity of Δ*MoMed15*, Δ*MoGcn5*, and Δ*MoSnf5* transformants to IPT. (B) Expression of MoPGR1 in Δ*MoSnf5* transformant

determined by RT-qPCR. The *MoActin* gene was used as the internal reference for normalization. Data presented are the mean ± SD (n = 3). Bars followed by the same letter are not significantly different according to a LSD test at P =0.01.
